# Fast and stable vapochromic response induced through nanocrystal formation of a luminescent platinum(II) complex on periodic mesoporous organosilica

**DOI:** 10.1038/s41598-019-51615-w

**Published:** 2019-10-22

**Authors:** Hiroki Matsukawa, Masaki Yoshida, Takahiro Tsunenari, Shunsuke Nozawa, Ayana Sato-Tomita, Yoshifumi Maegawa, Shinji Inagaki, Atsushi Kobayashi, Masako Kato

**Affiliations:** 1 Department of Chemistry, Faculty of Science, Hokkaido University, North-10 West-8, Kita-ku, Sapporo, Hokkaido 060-0810 Japan; 2 Institute of Materials Structure Science, High Energy Accelerator Research Organization (KEK), 1-1 Oho, Tsukuba, Ibaraki 305-0801 Japan; 3 Division of Biophysics, Department of Physiology, Jichi Medical University, 3311-1 Yakushiji, Shimotsuke, Tochigi 329-0498 Japan; 4 Toyota Central R&D Laboratories, Inc., Nagakute, Aichi 480-1192 Japan

**Keywords:** Materials chemistry, Coordination chemistry

## Abstract

A hybrid vapoluminescent system exhibiting fast and repeatable response was constructed using periodic mesoporous organosilica with bipyridine moieties (BPy-PMO) and a Pt(II) complex bearing a potentially luminescent 2-phenylpyridinato (ppy) ligand. An intense red luminescence appeared when the Pt(II)-complex immobilised BPy-PMO was exposed to methanol vapour and disappeared on exposure to pyridine vapour. The ON-OFF vapochromic behaviour occurred repeatedly in a methanol/pyridine/heating cycle. Interestingly, a rapid response was achieved in the second cycle and cycles thereafter. Scanning and transmission electron microscopies (SEM/TEM), absorption and emission, and nuclear magnetic resonance spectroscopies, mass spectrometry, and powder X-ray diffraction indicated that methanol vapour induced Si-C cleavage and thus liberated [Pt(ppy)(bpy)]Cl (bpy = 2,2′-bipyridine) from the BPy-PMO framework. Furthermore, the self-assembling properties of the Pt(II) complex resulted in the formation of highly luminescent micro/nanocrystals that were homogeneously dispersed on the porous support. The unique vapoluminescence triggered by the unprecedented protodesilylation on exposure to protic solvent vapour at room temperature is attributable to BPy-PMO being a giant ligand and an effective vapour condenser. Consequently, this hybrid system presents a new strategy for developing sensors using bulk powdery materials.

## Introduction

**Figure 1:**
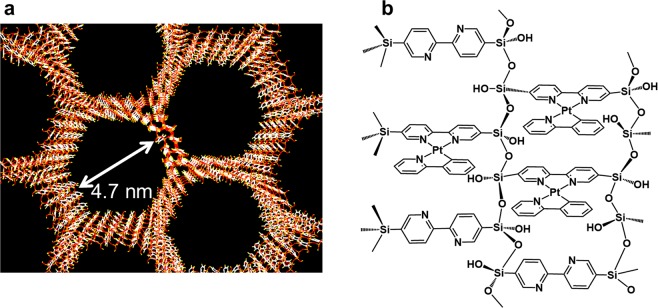
(**a**) Mesoporous structure of BPy-PMO. (**b**) Immobilised Pt complexes on BPy-PMO (**Pt-PMO**).

Porous materials are extremely attractive for the creation and control of nano- and meso-spaces where specific effects and novel phenomena are expected. Various nano- and mesoporous structures have been developed using inorganic, organic, and hybrid materials, which have been applied in catalytic systems, sensors, gas storage, electronic devices, and biological systems^1–3^. To build a mesoporous silica structure for luminescence, De Cola and co-workers developed mesoporous silica particles containing photofunctional metal complexes in their pores by using amphiphilic metal complexes as surfactants^4,5^. It was remarkable discovery that the confinement effect of mesoporous silica induced an enhancement of the luminescence intensity of the included metal complexes. In contrast, periodic mesoporous organosilicas (PMOs) are relatively new materials with uniformly distributed organic and inorganic moieties within their frameworks^6–9^. Compared with other hybrid porous materials such as mesoporous silica and metal-organic frameworks (MOFs), PMOs have an advantage in that they possess large regulated pores of several nano-meters in diameter (i.e. mesopores), which allow construction of particular chemical reaction fields. Inagaki and co-workers also developed a sophisticated mesoporous organosilica that incorporated various aromatic organic moieties^10^. In addition to the high stability and light-harvesting effect of the PMOs, assembly control of the functional metal complex systems was expected^11,12^. In particular, BPy-PMO, composed of silicate and 2,2′-bipyridine (bpy) connected by covalent bonds, allowed the metal ions to be captured through direct coordination to the periodic mesoporous framework (Fig. 1a)^13^. This PMO was then utilised as a photocatalytic system with polypyridine-Ru(II) and Ir(III), Re(I), Cu(II), and Pt(II) complexes as well as other selective catalytic systems^10,14–18^. However, the full potential of the periodic structure of this huge ligand (BPy-PMO) has not yet been studied.

Pt(II) complexes are known to exhibit a characteristic colour and luminescence when the square-planar complex units are stacked with Pt···Pt short contacts^19–21^. Thus, the colour and luminescence of such assembly-induced luminescent Pt(II) complexes should be extremely sensitive to changes in the stacking structures. External stimuli such as heat, pressure, vapour, and mechanical forces readily induce such changes. In particular, a vapour-induced reversible colour change, vapochromism, is a characteristic property of Pt(II)-complex assemblies^22–25^ and has attracted significant attention based on an easy sensing of the environment^26–28^. Various vapochromic systems have thus been developed using different metal complexes and organic crystals^29–35^. For vapochromic materials, however, the response rate to dilute gaseous molecules is an issue to be overcome in bulk systems, although it can be used to detect long-term changes like such as blue silica-gel as a moisture indicator. Aiming at a rapid response, various systems have recently been developed using MOFs^36,37^ and supramolecular porous crystals^33,38^, which have a large surface area directly accessible to the inner chromophores. The fabrication of thin films is an effective way to achieve a rapid response^39–41^. Meanwhile, soft materials have an advantage in terms of stimulus responsiveness and chemical sensing^42–46^. However, higher-order systems with less fluctuation are desired to improve the accuracy. In this context, a new category of promising stimulus-responsive materials that have both properties of soft and high-order materials, termed soft crystals, is proposed^47^. In addition, if the vapochromic properties are accompanied by changes in luminescence, a higher vapour-sensitivity will be possible. Therefore, the exploration of more sophisticated vapoluminescent systems having high sensitivity and selectivity remains a challenging subject.

In this study, we developed a unique vapochromic luminescent system that exhibited a rapid and stable vapour response with clear colour changes using integrated coordination sites and the vapour absorptivity of BPy-PMO. By immobilisation of a Pt(II) complex on BPy-PMO with a high ratio, the [{Pt(ppy)}_*n*_(BPy-PMO)]Cl_*n*_ (ppy = 2-phenylpyridinate, Fig. 1b) (**Pt-PMO**) system successfully achieved vapour-induced nanocrystal formation and repeatable vapour response.

## Results

### Synthesis and characterisation

**Figure 2:**
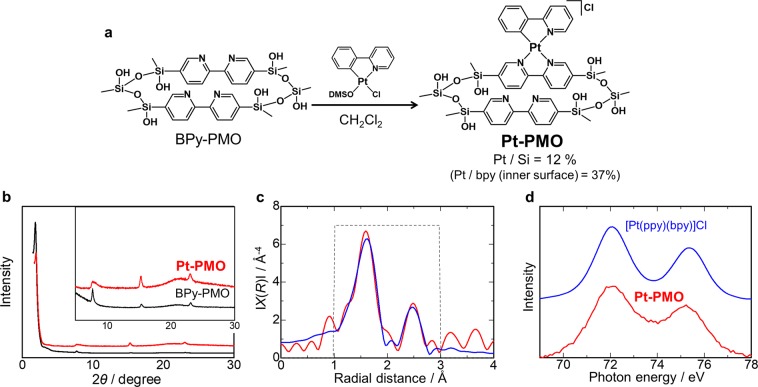
(**a**) Synthetic scheme of **Pt-PMO**. (**b**) PXRD patterns of **Pt-PMO** (red line) and BPy-PMO (black line). (**c**) EXAFS Fourier transform of Pt-L_III_ edge of **Pt-PMO**. The experimental data, the fitting curve of the data, and the Hanning window are shown as red, blue, and broken gray lines, respectively. A fitting analysis of the EXAFS data was conducted based on the analysed crystal structure of [Pt(bpy)(ppy)](PF_6_) (see Fig. S3). (**d**) XPS spectra of **Pt-PMO** (red line) and [Pt(bpy)(ppy)]Cl (blue line). The background was subtracted using the Shirley method. The binding energy and the full width at half-maximum of each peak are summarised in Table S1.

Pt(II)-immobilised BPy-PMO (**Pt-PMO**) with a favourable immobilisation amount was successfully obtained by reacting BPy-PMO with [Pt(ppy)Cl(DMSO)] (DMSO = dimethyl sulfoxide) in CH_2_Cl_2_ instead of DMSO, which was used in our previous report (Fig. 2a)^16^. The immobilised amount was estimated to be 12% based on the Pt/Si ratio using X-ray fluorescence (XRF) spectroscopy and UV-Vis absorption spectra (Fig. S1). The powder X-ray diffraction (PXRD) pattern of **Pt-PMO** clearly showed characteristic peaks at 2*θ* = 1.84° (*d* = 4.8 nm), 7.7°, 15.4°, and 23.0° after the immobilisation, indicating that the ordered mesoporous structure and pore wall structure of BPy-PMO was maintained even after the immobilisation of the platinum(II) complex (Fig. 2b). To discuss the local structure around the Pt sites in **Pt-PMO**, the extended X-ray absorption fine structure (EXAFS) spectra were measured. The *k*^3^-weighted EXAFS spectrum of the Pt-L_III_ edge of **Pt-PMO** and the corresponding Fourier transform are shown in Figs S2a and 2c, respectively. The two broad peaks at ~1.4–1.9 Å and 2.2–2.7 Å were well-explained by the local structure of the first and second shells around the Pt site in the model complex [Pt(ppy)(bpy)](PF_6_), whose crystal structure was elucidated by single crystal X-ray analysis of a linear chain structure of the Pt(II) complex units with a moderate Pt···Pt distance (3.6048(1) Å) at 93 K (Fig. S3). The X-ray absorption near-edge spectrum (XANES) of **Pt-PMO** was also in good agreement with that of [Pt(ppy)(bpy)](PF_6_) (Fig. S2b). The X-ray photoelectron spectroscopy (XPS) revealed that the binding energies of Pt 4f_7/2_ and Pt 4f_5/2_ signals of **Pt-PMO** (72.3 and 75.1 eV) were almost identical to those of the model complex [Pt(ppy)(bpy)]Cl (72.1 and 75.3 eV) (Fig. 2d, Table S1), which further supported the desired complexation in the mesopores of BPy-PMO.

The nitrogen adsorption isotherm of **Pt-PMO** revealed that the amount of adsorbed nitrogen was lower in comparison with BPy-PMO (Fig. S4). The Brunauer-Emmett-Teller (BET) surface area (*S*_BET_) decreased to 393 m^2^g^−1^ for **Pt-PMO** from 680 m^2^g^−1^ for BPy-PMO, and the non-linear density functional theory (NLDFT) analysis also revealed that the pore diameter of **Pt-PMO** (3.9 nm) was smaller than that of BPy-PMO (4.7 nm), indicating that the platinum(II) complex was immobilised in the mesopores (Table S2). Using the average particle diameter (695 nm) determined through dynamic light scattering (DLS) measurements and the pore diameter of BPy-PMO (4.7 nm), the ratio of the inner and outer surfaces area of the mesopore of BPy-PMO was roughly estimated to be approximately 105 (Fig. S5), which meant that 99% of the surface was inside the BPy-PMO. Assuming a three-layered structure of the wall and a full occupation of the outer surface bpy moieties by the Pt complexes^13^, 37% of the bpy moieties on the inner surface of the BPy-PMO were estimated to be occupied by the Pt-ppy units for the present **Pt-PMO** sample with 12% Pt/Si (Fig. S5).

**Figure 3:**
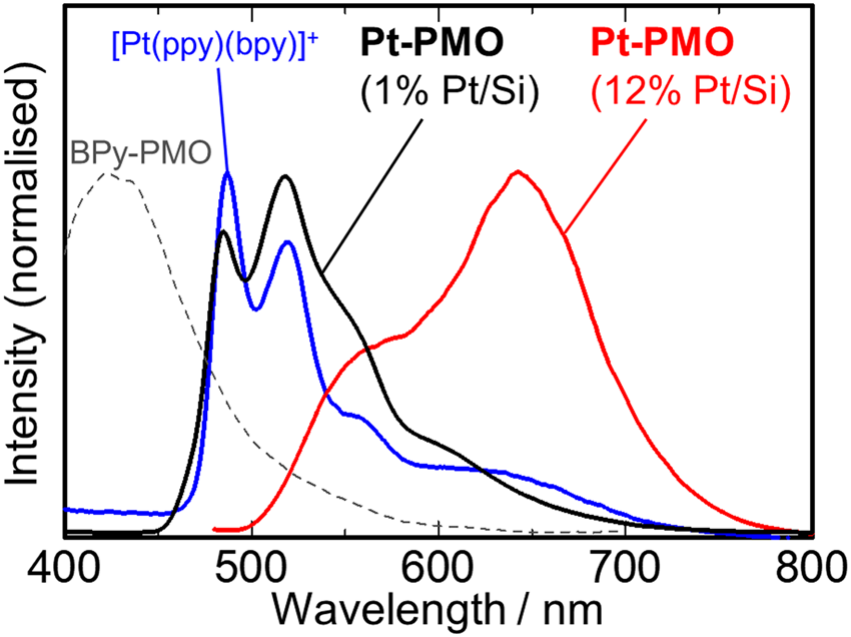
Emission spectra of **Pt-PMO** with an immobilised ratio of 12% Pt/Si (red line) and 1% Pt/Si (black line) at 77 K, BPy-PMO (gray broken line) at 77 K, and [Pt(ppy)(bpy)]Cl (blue line) in methanol at 298 K. Excitation wavelengths in all cases was 410 nm except for BPy-PMO (380 nm).

The emission properties of **Pt-PMO** provide useful information regarding the assembly of the Pt(II) complex units. **Pt-PMO** with 12% Pt/Si exhibited a broad emission spectrum at 77 K (Fig. 3, Table S3). This is in contrast to that for **Pt-PMO** with its small loading amount of the platinum complex (1% Pt/Si) at 77 K, which showed a typical ^3^ππ* emission with a vibronic structure similar to that for the model complex [Pt(ppy)(bpy)]^+^ in a dilute methanol solution (Fig. 3). Considering that there was no change in the diffuse reflectance spectra of 1% and 12% **Pt-PMO** (Fig. S6), the broad luminescence for 12% **Pt-PMO** was attributable to the emissions from the excimeric or dimeric form of platinum complexes units. In fact, the excimer emission of another PMO incorporating biphenyl (Bp-PMO) has been reported previously^48,49^. Additionally, an emission at ~450 nm was observed in the system of BPy-PMO, which is completely different from that observed for 12% **Pt-PMO** (Fig. 3). This is an important point because the broad emission indicates an assembled immobilisation of the Pt(II) complex units in the meso-pore that allows the electronic interactions between the Pt(II) complex units in the excited states.

### Vapochromic response

**Figure 4:**
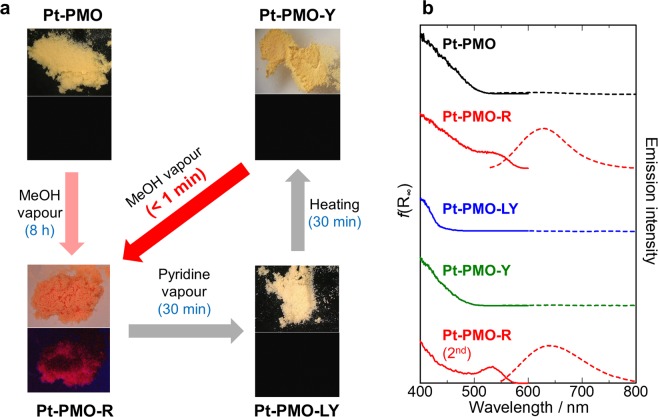
(**a**) Vapour response cycle of **Pt-PMO**. The upper photos are images under daylight, and the lower photos are those under UV light. Each photograph is 2.0 mm × 1.6 mm in size. (**b**) Changes in the UV-Vis diffuse reflectance spectra (solid lines) and emission spectra (broken lines) during the vapour response cycle at room temperature. Excitation wavelengths in all cases was 410 nm except for **Pt-PMO-R** (450 nm), which provided the same emission spectrum by the excitation at either wavelength. For the emission and excitation spectra at 77 K, see also Fig. S12.

Interestingly, **Pt-PMO** exhibited a distinct vapochromic behaviour. The yellow powder sample of **Pt-PMO** (12% Pt/Si) turned into a reddish colour upon exposure to MeOH vapour, accompanied by the appearance of a new band at 530 nm, as shown in the diffuse reflectance spectra (Fig. 4). Simultaneously, a broad emission band appeared with a maximum at 630 nm, and the emission intensity reached the maximum level after 8 h under nearly saturated vapour pressure at room temperature (Fig. S7). This emission band at 630 nm and the quantum yield (*Φ*) of 0.11 were consistent with that observed for the model complex [Pt(ppy)(bpy)]Cl in the solid state (*Φ* = 0.12 and *τ* = 123 ns at 298 K, as shown in Table S3), suggesting that the emission is originated from the triplet metal-metal-to-ligand charge transfer (^3^MMLCT) in an excited state arising from the Pt···Pt electronic interaction^19,22^. In fact, the red emission of both the orange powdery sample (called **Pt-PMO-R**) and the crystalline sample of [Pt(ppy)(bpy)]Cl were red-shifted by 35–40 nm with sharpening at 77 K indicating typical spectral features of ^3^MMLCT emissions (Figs S8 and S9). These results suggest that a regularly assembled form of the Pt(II) complex units was formed on the BPy-PMO upon exposure to the MeOH vapour.

Next, **Pt-PMO-R** was heated at 403 K for 12 h to remove the MeOH molecules (Fig. S10a). The emission band did not change even after the removal of MeOH (Fig. S10b), which suggested an excellent stability of **Pt-PMO-R**. However, when it was exposed to the pyridine vapour, its colour readily changed from red to light yellow (hereafter, called **Pt-PMO-LY**) within ~10 min, demonstrating a considerable blue-shift (~140 nm) of the absorption edge (Fig. 4b). Along with the absorption spectral change, the emission intensity decreased and disappeared eventually (Fig. S11a). At 77 K, **Pt-PMO-LY** exhibited a green emission at *λ*_max_ = 481 nm with a clear vibrational progression (Fig. S12) attributable to the ligand-based ^3^ππ* emission, similar to that for the model complex [Pt(ppy)(bpy)]^+^ in a dilute methanol solution (Table S3), indicating the negligible Pt···Pt interactions in this form in contrast to those of **Pt-PMO-R**. On heating the **Pt-PMO-LY** form at 353 K for 30 min for the removal of the pyridine vapour, it returned to a yellow powder (called **Pt-PMO-Y**), showing almost the same absorption spectrum as the **Pt-PMO** form (Fig. 4b).

**Figure 5:**
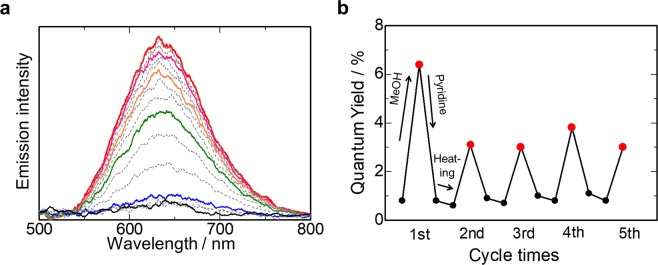
(**a**) Evolution of the emission spectrum of **Pt-PMO-Y** upon MeOH vapour exposure at 323 K (*λ*_ex_ = 410 nm). The spectra were recorded at 20-s intervals (the solid lines show an increment of 1 min). It should be noted that the induction period (ca. 1 min) corresponds to the time required to reach the methanol vapour through diffusion (see Fig. S13). (**b**) Reversible on-off cycles monitored based on luminescence quantum yield (*λ*_ex_ = 410 nm). The red dots indicate an “ON” state after MeOH vapour exposure (approximately 1-min exposure except for 1 h for the first cycle), and the black dots indicate “OFF” states after pyridine vapour exposure and heating.

Surprisingly, the vapochromic response of **Pt-PMO-Y** was drastically accelerated as compared with that of the as-synthesised **Pt-PMO**. Upon exposure to methanol vapour, the colour of **Pt-PMO-Y** rapidly turned from yellow to red within tens of seconds and the **Pt-PMO-R** form was obtained again. Along with this change in colour, the broad emission band at 630 nm also increased rapidly upon exposure to methanol vapour (Fig. 5a). Subsequently, exposing **Pt-PMO-R** (second cycle) to pyridine vapour resulted in the regeneration of the **Pt-PMO-LY** form again. This three-state vapochromic cycle of **Pt-PMO-R**/**Pt-PMO-LY**/**Pt-PMO-Y** showed a high reversibility, as indicated in Fig. 5b, which was pursued through changes in the emission quantum yield.

Furthermore, the present system also enabled the vapochromic response in a low relative pressure (*P*/*P*_0_) region because of the mesoporous structure. **Pt-PMO-Y** showed a vapochromic luminescence even at *P*/*P*_0_ = 0.1 (Fig. S14). As previously reported, the pore walls of BPy-PMO contains a considerable amount of silanol (Si-OH) groups^10^, which is expected to capture methanol through hydrogen bonding even at low relative pressure, in addition to the capillary condensation effect in the mesoporous channel. Indeed, thermogravimetric (TG) analysis revealed that BPy-PMO can adsorb a considerable amount of methanol molecules, whereas almost no adsorption was observed after the protection of silanol by trimethylsilyl groups (Fig. S10a). The methanol vapour adsorption isotherm (Fig. S15) showed a large amount of methanol adsorption at a very low relative pressure (~20 mol·mol^−1^ per bpy unit at *P*/*P*_0_ = 0.1), and a significant hysteresis within the region of *P*/*P*_0_ > 0.6. Therefore, the high affinity of BPy-PMO for methanol was responsible for the low detection limit of **Pt-PMO-Y**, and the fast vapochromic response.

### Vapochromic mechanism

**Figure 6:**
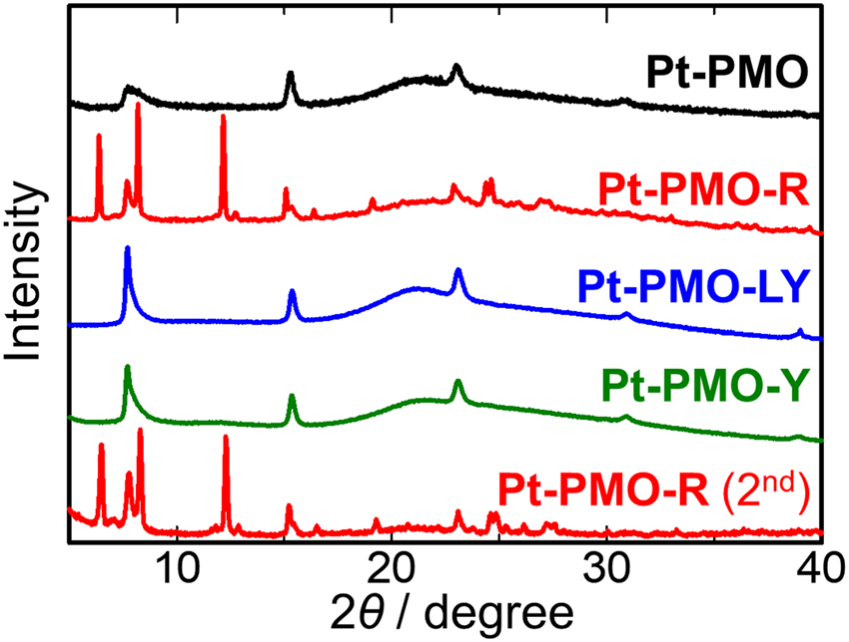
PXRD patterns of **Pt-PMO** during vapour response cycles.

To investigate the origin of this vapochromism in detail, we used microscopic techniques and PXRD to observe the morphological changes occurring in the vapochromic cycle. Interestingly, several new diffraction peaks appeared only for the **Pt-PMO-R** stage (red line in Fig. 6) in addition to those for the periodic structure of BPy-PMO, which indicated the formation of a new crystalline species on BPy-PMO through the methanol vapour exposure. After exposure to pyridine vapour, these crystalline peaks disappeared, with the BPy-PMO peaks remaining (blue line in Fig. 6); essentially the same patterns were also observed after heating (orange line in Fig. 6). The diffraction peaks for **Pt-PMO-R** were regenerated using the second methanol vapour exposure (lower red line in Fig. 6).

**Figure 7:**
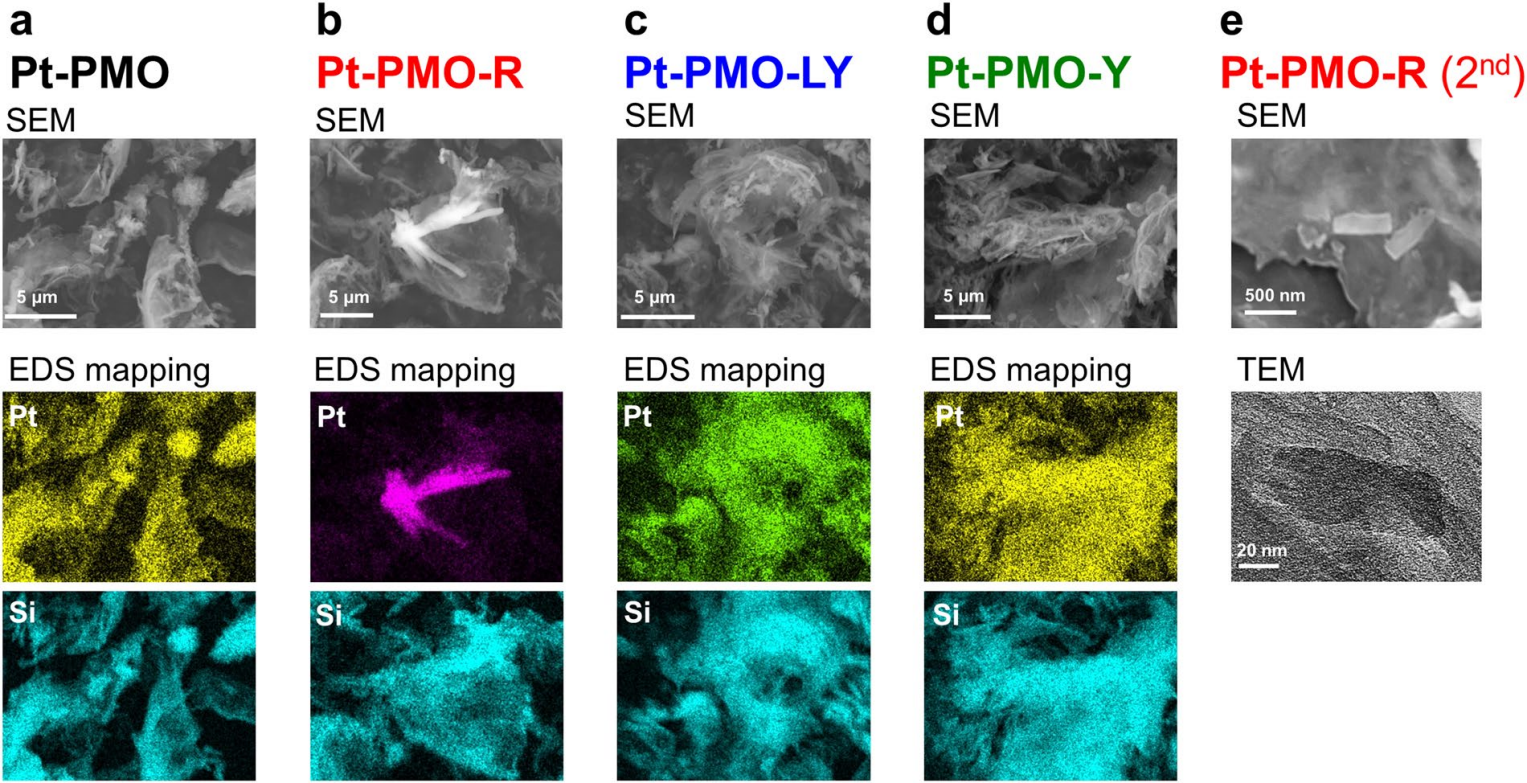
SEM or TEM images of (**a**) **Pt-PMO**, (**b**) **Pt-PMO-R**, (**c**) **Pt-PMO-LY**, (**d**) **Pt-PMO-Y**, and (**e**) **Pt-PMO-R** (2^nd^).

The scanning and transmission electron microscopic (SEM and TEM, respectively) observations with energy dispersive X-ray spectrometry (EDS) clearly indicated the morphological changes (Figs 7 and S16). Initially, no crystalline species were observed on the **Pt-PMO** particles, and the EDS elemental map showed that Pt atoms were homogeneously immobilised on BPy-PMO (Figs 7a and S16a). After methanol vapour exposure (i.e. the formation of **Pt-PMO-R**), the SEM image showed that crystals with a length of 1–10 μm appeared on the BPy-PMO substrate (Fig. 7b), as suggested by the PXRD data. The EDS elemental map clearly showed that Pt atoms were mainly localised in the crystals (Fig. 7b), and the EDS single-point analysis suggested that crystals of a Pt(II) complex were generated (Fig. S16b). Such crystalline materials were observed neither for **Pt-PMO-LY** (Fig. 7c) nor **Pt-PMO-Y** (Fig. 7d). After the second methanol vapour exposure, crystalline materials were observed again. However, in contrast to the first observation, these crystals were much smaller with a length of 80–400 nm (Fig. 7e). These nano-sized crystals were confirmed to be those of a Pt(II) complex through EDS single-point analysis and TEM (Fig. S16e). The formation of such nanocrystals is likely the reason for the fast vapochromic response during the second cycle, as discussed in the next section.

To identify this crystalline species on **Pt-PMO-R**, mass spectrometry (MS) and NMR analyses were conducted. Importantly, both ESI-MS for the soluble species of **Pt-PMO-R** and MALDI-MS for the powdery sample of **Pt-PMO-R** showed the main signal at *m*/*z* = 505.1 (Fig. S17), which is consistent with the Pt(II) complex ion, {Pt(ppy)(bpy)}^+^, including an isotropic peak pattern. The ^1^H NMR spectrum of the extracted species from **Pt-PMO-R** with methanol was also identical to that of [Pt(ppy)(bpy)]Cl (Fig. S18). These results suggest that [Pt(ppy)(bpy)]^+^ was formed through the Si–C(bpy) bond dissociation from the BPy-PMO framework. It is notable that the double Si–C bond dissociations occurred under such mild conditions through methanol vapour exposure considering that BPy-PMO is a known stable framework and Si–C bond dissociations typically occur only under the strongly acidic or basic conditions^13,50^. An immobilised Pt complex may allow a nucleophilic attack of methanol molecules on BPy-PMO (i.e. protodesilylation)^51^ while maintaining the entire framework of BPy-PMO. This vapour-triggered protodesilylation process was further evidenced through the deuterium-labelling experiments, in which the deuterated complexes, [Pt(ppy)(bpy-d_2_)]^+^ and [Pt(ppy)(bpy-d_1_)]^+^, were obtained when **Pt-PMO** was exposed to methanol-d_4_ vapour, and confirmed through ESI-MS and ^1^H NMR (Figs S19 and S20). Based on these results, we concluded that the methanol-vapour induced crystal formation of [Pt(ppy)(bpy)]Cl occurred through Si–C bond dissociation in **Pt-PMO-R**. Indeed, the diffraction peaks of the crystalline species of **Pt-PMO-R** qualitatively agreed with those of the model complex [Pt(ppy)(bpy)]Cl in a methanol atmosphere (Fig. S21). The XRF spectra indicated that ~2/3 of the immobilised Pt(II) complex was detached to form crystals through vapour-induced protodesilylation (Fig. S22). This conclusion was further supported by the fact that the BET surface area and average pore diameter of **Pt-PMO-R** (420 m^2^g^−1^ and 4.2 nm, respectively, as shown in Fig. S4 and Table S2) definitively increased from those of **Pt-PMO** (393 m^2^g^−1^ and 3.9 nm, respectively) owing to the detachment of [Pt(ppy)(bpy)]^+^ ions through a vapour-triggered Si–C bond dissociation.

**Pt-PMO** also exhibited a vapochromic response to other protic vapours such as H_**2**_O, EtOH, and *i*-PrOH, as indicated from the emission spectra and PXRD patterns, although it did not respond to less-polar vapours such as CH_2_Cl_2_, chloroform, and toluene (Figs S23 and S24). In addition, it is also noteworthy that the vapour-triggered protodesilylation is characteristic of metal-loaded BPy-PMOs and never occurred for a discrete Pt(II) complex bearing a precursor unit of BPy-PMO in the same conditions (Figs S25 and S26).

## Discussion

**Figure 8:**
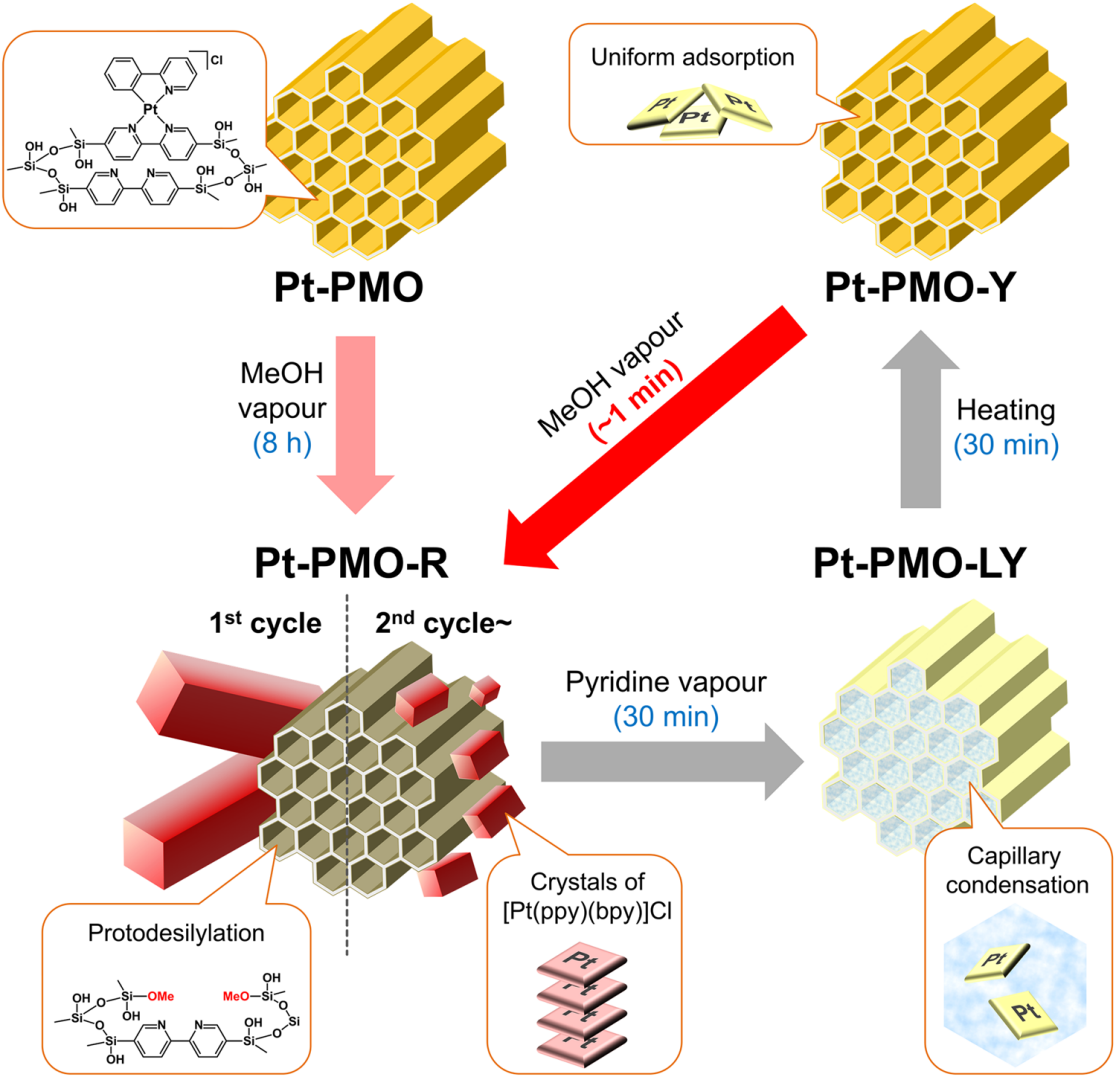
Schematic illustration of the vapour response cycle.

The structural, spectroscopic, and microscopic investigations of the interesting behaviours of the mesoporous materials revealed not only the vapochromic mechanism but also the reason for the fast response. A schematic illustration of this vapour response cycle is shown in Fig. 8.

Before the vapour exposure, the Pt(II) complex was homogeneously and densely immobilised on BPy-PMO through the coordination bonds. Based on the first methanol vapour exposure, however, the Si–C bonds on the immobilised Pt(II) complexes were cleaved using methanol vapour to form micrometre-sized crystals of [Pt(ppy)(bpy)]Cl, leading to a change in colour to red (i.e. **Pt-PMO-R**), as confirmed through isotropic experiments. The micrometre-sized crystals of [Pt(ppy)(bpy)]Cl on the BPy-PMO were dissolved in pyridine upon exposure to pyridine vapour, and uniformly adsorbed in the mesopores of BPy-PMO through the capillary condensation, as evidenced by SEM-EDS, resulting in a light yellow colour (i.e. **Pt-PMO-LY**) of the discrete molecules of the Pt(II) complex without any intermolecular interactions. This is in contrast to the lack of change in the luminescence spectrum when exposed to acetonitrile vapour instead of pyridine vapour (Fig. S11b), probably because of a weaker coordination ability of acetonitrile as compared to that of pyridine. After the removal of pyridine through heating, the Pt(II) complex was uniformly loaded onto BPy-PMO using physisorption without any Pt···Pt interaction (i.e. **Pt-PMO-Y**), as suggested by the PXRD pattern (Fig. 6). It is interesting to note that the nanocrystals of [Pt(ppy)(bpy)]Cl were produced from **Pt-PMO-Y** on exposure to methanol vapour. This is in contrast to the recrystallisation of [Pt(ppy)(bpy)]Cl from a normal pyridine solution, which fails because of the decomposition of the complex (Fig. S27). The meso-space of BPy-PMO must also be effective also for the crystallisation. It is reasonable for the vapochromic response in the second and subsequent exposures to be much faster than during the first exposure because the Si-C bond cleavage is no longer necessary. In addition, it should be noted that a uniform loading of [Pt(ppy)(bpy)]Cl is key to obtaining such a fast and clear vapochromic response. We were unable to obtain similar results by using a mixture of [Pt(ppy)(bpy)]Cl and BPy-PMO instead of **Pt-PMO** (Fig. S28).

For many vapochromic materials, the vapour response typically requires several hours to days in a bulk state. In addition, vapochromic materials generally suffer from poor stability in the vapour-included forms, and quickly restore the original colour through the desorption of vapour molecules^22–25^. Stable and as-desired changeable systems are necessary for perfectly controlled vapochromic systems. It is noteworthy that the present three-step vapochromic system **Pt-PMO** enables a rapid response within several tens of seconds through exposure to methanol vapour in a bulk state accompanied by an ON-OFF switching of the emission. In addition, once **Pt-PMO-R** is formed in response to methanol vapour, it demonstrates high stability after its removal, maintaining the “vapour-detection history,” which has been difficult to observe thus far except for a few examples^52,53^. In addition, it is also extremely easy to erase the history (switch-off) through pyridine vapour exposure.

In conclusion, we successfully constructed and elucidated a superior vapochromic system utilising a cooperative phenomenon arising from a huge mesoporous ligating support, BPy-PMO, with an assembly-induced Pt(II) complex. Our findings provide a new guiding principle for the development of photofunctional materials.

## Methods

### Materials

Reagents and solvents were purchased from commercial sources and used without further purification. BPy-PMO^13^, trimethylsilyl-protected BPy-PMO^13^, **Pt-PMO** (1% Pt/Si)^16^, and [Pt(ppy)Cl(DMSO)]^54^ were prepared as previously described.

### Synthesis of model complexes, [Pt(ppy)(bpy)]X (X = Cl^−^)

Although the synthetic procedure of [Pt(ppy)(bpy)]Cl has been previously reported^16,55^, in this study, it was prepared using another method that is similar to the immobilisation of the Pt(II) complex. A solution of [Pt(ppy)Cl(DMSO)] (117 mg, 0.25 mmol) and bpy (43 mg, 0.27 mmol) in CH_2_Cl_2_ (15 mL) was stirred at room temperature for 5 h. The red precipitate was collected through filtration, washed with CH_2_Cl_2_, methanol, and diethyl ether, and dried *in vacuo*. Yield: 125 mg (0.23 mmol, 89%). Elemental analysis, calculated for C_21_H_16_N_3_ClPt·0.75H_2_O: C: 45.49, H: 3.18, N: 7.48. Found: C: 45.68, H: 3.21, N: 7.34. ^1^H NMR (400 MHz, methanol-*d*_4_, δ): 9.46 (d, 1H, *J* = 5.6 Hz), 9.10 (d, 1H, *J* = 5.7 Hz), 8.90 (d, 1H, *J* = 5.7 Hz), 8.63 (d, 1H, *J* = 7.9 Hz), 8.57 (d, 1H, *J* = 7.9 Hz), 8.42 (m, 2H), 8.14 (td, 1H, *J* = 7.4 Hz, 1.2 Hz), 8.07 (d, 1H, *J* = 7.4 Hz), 7.94 (td, 1H, *J* = 6.6 Hz, 1.1 Hz), 7.84 (td, 1H, *J* = 6.7 Hz, 1.0 Hz), 7.73 (dd, 1H, *J* = 7.5, 1.0 Hz), 7.48 (td, 1H, *J* = 6.7 Hz, 1.3 Hz), 7.37 (d, 1H, *J* = 8.1 Hz), 7.29 (td, 1H, *J* = 7.3 Hz, 1.3 Hz), 7.22 (td, 1H, *J* = 7.3 Hz, 1.3 Hz). ESI-MS (methanol, positive): *m/z* 505.1 ({[Pt(ppy)(bpy)]}^+^). **(X = PF**_**6**_^**−**^**)** [Pt(ppy)(bpy)]Cl (110 mg, 0.203 mmol) was dissolved in a CH_3_CN solution at 70 °C followed by the addition of AgPF_6_ (51.4 mg, 0.203 mmol). The reaction solution was refluxed by stirring in the dark for 3 h and filtered through Celite for the removal of AgCl, followed by evaporation under reduced pressure. The orange precipitate was collected through filtration, washed with a small amount of CH_2_Cl_2_ and Et_2_O, and dried in vacuo. Single crystals suitable for X-ray crystallography were obtained through the slow vapour diffusion of CH_2_Cl_2_ into a solution of [Pt(ppy)(bpy)](PF_6_) in a minimum amount of THF at 0 °C. Red needle-like crystals of [Pt(ppy)(bpy)](PF_6_) were separated after several days. ^1^H NMR (270 MHz, DMSO-*d*_6_, δ): 9.51 (d, 1H, *J* = 6.0 Hz), 8.69 (d, 2H, *J* = 4.7 Hz), 8.39 (d, 2H, *J* = 8.0 Hz), 8.22 (d, 1H, *J = *6.8 Hz), 8.15 (m, 2H), 7.95 (t, 2H, *J* = 8.0 Hz), 7.79 (d, 1H, *J* = 7.5 Hz), 7.51 (t, 1H, *J* = 6.0 Hz), 7.45 (m, 2H), 7.16 (t, 2H, *J* = 7.5 Hz).

### Preparation of higher-loaded Pt-immobilised BPy-PMO (Pt-PMO)

A suspension of BPy-PMO (107 mg, 0.40 mmol as a repeating unit) and [Pt(ppy)Cl(DMSO)] (171 mg, 0.40 mmol) in CH_2_Cl_2_ (10 mL) was refluxed for 3 h. The yellow powdery solid was collected through filtration, washed with CH_2_Cl_2_, acetone, and Et_2_O, and dried* in* *vacuo*. Yield: 125 mg. (Immobilised ratio based on bpy in PMO: 12% Pt/Si based on XRF).

### Measurements

Elemental analyses and electrospray-ionisation mass spectrometry (ESI-MS) were conducted at the analysis centre of Hokkaido University. The ^1^H NMR and ^1^H-^1^H COSY NMR spectra were acquired on a JEOL ECZ-400S or EX-270 spectrometer. Energy dispersive XRF spectra were acquired on a JEOL JSX-3100RII spectrometer using a Rh target. DLS analyses were conducted using an OTSUKA ELSZ-1000SCl analyser. Nitrogen and methanol vapour adsorption isotherms were measured using an automatic BELSORP-max (MicrotracBEL Co.) volumetric adsorption apparatus. Pore-size distributions were calculated using the density functional theory (DFT) method (DFT kernel: N_2_ at 77 K on silica, cylindrical pores, and NLDFT equilibrium model). Thermogravimetry-differential thermal analysis (TG-DTA) measurements were recorded using a Rigaku Thermoplus EVO TG-DTA 8120 with Al sample pans under an Ar flow. Matrix-assisted laser desorption/ionisation mass spectrometry (MALDI-MS) was conducted using a Bruker Microflex LRF spectrometer in a linear mode.

### Electron microscopy

TEM was performed on a JEOL JEM-2010 FASTEM microscope at an accelerating voltage of 200 kV. Field-emission scanning electron microscopy (FE-SEM) using EDS was conducted on a JEOL JSM-7100F microscope at an accelerating voltage of 15 kV equipped with an electron backscatter diffraction detector (Oxford Aztec Energy-HKL).

### Powder X-ray diffraction

PXRD measurements were conducted using Cu *K*_α_ radiation (λ = 1.5418 Å) on a Bruker D8 Advance diffractometer equipped with a graphite monochromator and a one-dimensional LinxEye detector, or a Rigaku SPD diffractometer at the BL-8B beamline of the Photon Factory (PF), Japan. The synchrotron X-ray wavelength was 1.5455 Å.

### X-ray absorption fine structure measurements

Pt-L_III_-edge EXAFS spectra were collected in the florescence mode at BL-12C beamline of the PF, Japan. The incident X-ray was made monochromatic using a Si(111) double-crystal monochromator. *k*^3^-weighted EXAFS function, *k*^3^*χ*(*k*), was extracted from the raw X-ray absorption data, which were obtained using the ATHENA software^56^. A Fourier transform of the EXAFS function was conducted within the *k* range of 2–14 Å^−1^ using a Hanning window, and it was fit to the **Pt-PMO** structure using ARTEMIS software^56^ in the range (*R*) of 1–3 Å corresponding to the first and second shells. In this fitting, the amplitude and phase shift for all scattering paths were also calculated using FEFF6L in the ARTEMIS software^56^.

### X-ray photoelectron spectroscopy

XPS were recorded on a JEOL JPC-9010MC spectrometer at a vacuum pressure of less than 1 × 10^−5^ Pa in an analysis chamber. A standard Al *K*_α_ excitation source (1486.6 eV) was used during all experiments. Each sample was placed on carbon tape. The binding energies were calibrated against the carbon 1s (284.6 eV) peak position. A spectral fitting was conducted using a Gaussian-Lorentzian product function.

### Single Crystal X-ray crystallography

Diffraction data for [Pt(ppy)(bpy)](PF_6_) were collected using a Rigaku XtaLAB-PRO diffractometer with a Hypix-6000HE area detector and a multilayer mirror-monochromated Cu *K*_α_ radiation (*λ* = 1.54184 Å) at 93 K. The crystal was mounted in a microloop with Paratone-N oil. Diffraction data were collected and processed using CrysAlisPro^57^ at 93 K. The structures were solved using a SHELXT^58^ structure solution program applying intrinsic phasing, and refined using the SHELXL^58^ refinement package with least squares minimisation. Because the solvent molecules in the unit cell were significantly disordered and could not be properly modelled, the solvent mask routine implemented in Olex2^59^ was used to calculate the solvent disorder area and remove its contribution to the overall intensity data. All non-hydrogen atoms were refined anisotropically and H atoms were refined using the riding model. The crystallographic data are summarised in Table S4 and have been deposited at the Cambridge Crystallographic Data Centre (CCDC 1910647). The molecular and stacking structures are shown in Fig. S3, and selected bond distances and angles are summarised in Table S5.

### Absorption and emission measurements

UV-Vis absorption spectra were recorded on a Shimadzu UV-2500PC spectrophotometer. UV-Vis diffuse reflectance spectra were obtained using the same spectrophotometer equipped with an integrating sphere apparatus. The reflectivity of the solid samples was converted using the Kubelka-Munk function. Emission spectra were recorded on a JASCO FP-8600 spectrophotometer. Luminescence quantum yields were recorded on a Hamamatsu Photonics C9920-02 absolute photoluminescence quantum yield measurement system equipped with an integrating sphere apparatus and a 150 W CW xenon light source. The accuracy of the instrument was confirmed based on a measurement of the quantum yield of anthracene in ethanol (*Φ* = 0.27)^60^. The emission lifetime measurements were conducted using a Hamamatsu Photonics Quantaurus-Tau C11367 system.

## Supplementary information

Supplementary data
