# Supplementary material for: Fast and stable vapochromic response induced through nanocrystal formation of a luminescent platinum(II) complex on periodic mesoporous organosilica
Source: Sci Rep. 2019 Oct 22;9:15151. doi: 10.1038/s41598-019-51615-w (PMC6806002; doi:10.1038/s41598-019-51615-w)
Supplement: Supplementary file 1 — Supplementary data [file 41598_2019_51615_MOESM1_ESM.pdf]

Supplementary information for

Fast and stable vapochromic response induced through nano-crystal formation of a luminescent platinum(II) complex on periodic mesoporous organosilica

Hiroki Matsukawa,[a] *Masaki Yoshida,*[a] *Takahiro Tsunenari,*[a] *Shunsuke Nozawa,*[b] *Ayana Sato-Tomita,*[c] *Yoshifumi Maegawa,*[d] *Shinji Inagaki,*[d] *Atsushi Kobayashi,*[a] *and Masako Kato*[a,*]

[a] Department of Chemistry, Faculty of Science, Hokkaido University, North-10 West-8, Kita-ku, Sapporo, Hokkaido 060-0810, Japan.

[b] Institute of Materials Structure Science, High Energy Accelerator Research Organization (KEK), 1-1 Oho, Tsukuba, Ibaraki 305-0801, Japan.

[c] Division of Biophysics, Department of Physiology, Jichi Medical University, 3311-1 Yakushiji, Shimotsuke, Tochigi 329-0498, Japan.

[d] Toyota Central R&D Laboratories, Inc., Nagakute, Aichi 480-1192, Japan.

[*] Corresponding Author:
Masako Kato
E-mail: mkato@sci.hokudai.ac.jp

Table of Contents

Fig. S1 Determination of immobilized ratio of Pt(II) complex on PMO.
Fig. S2 EXAFS and XANES spectra of **Pt-PMO**.
Fig. S3 Crystal structure of [Pt(ppy)(bpy)](PF_6).
Fig. S4 Nitrogen adsorption isotherms of BPy-PMO, **Pt-PMO**, and **Pt-PMO-R**.
Fig. S5 Particle diameter distributions of BPy-PMO estimated by the DLS.
Fig. S6 UV-vis diffuse reflectance spectra of **Pt-PMO**.
Fig. S7 Changes of the emission spectrum of **Pt-PMO** during methanol vapour exposure.
Fig. S8 Emission spectra of [Pt(ppy)(bpy)]Cl and [Pt(ppy)(bpy)](PF_6) in the solid state.
Fig. S9 Emission and excitation spectra of **Pt-PMO**.
Fig. S10 (a) TG of BPy-PMO and **Pt-PMO**, and (b) emission spectra of **Pt-PMO-R** before and after removal of methanol by heating.
Fig. S11 Changes of the emission spectrum of **Pt-PMO-R** during pyridine vapour exposure (a) and acetonitrile vapour (b).
Fig. S12 Emission spectral change of **Pt-PMO** at 77 K during the vapour response cycle.
Fig. S13 The experimental setup for the measurement of emission spectral changes under vapour exposure.
Fig. S14 Emission spectra of **Pt-PMO-Y** after the exposure to various relative pressures of methanol vapour.
Fig. S15 Methanol vapour adsorption isotherms of BPy-PMO, **Pt-PMO**, and **Pt-PMO-Y**.
Fig. S16 SEM images, EDS elemental mapping, and TEM images.
Fig. S17 MALDI-MS and ESI-MS of **Pt-PMO** and **Pt-PMO-R**.
Fig. S18 ^{1}H NMR spectrum of the soluble species of **Pt-PMO-R**.
Fig. S19 ESI-MS of the soluble species of **Pt-PMO** after the methnaol-d_4 vapour exposure.
Fig. S20 ^{1}H NMR spectrum of the soluble species of **Pt-PMO** after the methnaol-d_4 vapour exposure.
Fig. S21 PXRD patterns of **Pt-PMO-R** and [Pt(ppy)(bpy)]Cl.
Fig. S22 XRF spectrum of **Pt-PMO-R** before and after the extraction of the soluble species.
Fig. S23 Emission spectra of **Pt-PMO** after the exposure to several vapours.
Fig. S24 PXRD patterns of **Pt-PMO** after the exposure to several vapours.
Fig. S25 ^{1}H NMR spectra of (a) [$PtCl_2$(bpy)] and (b) the soluble species of **$PtCl_2$-PMO** after the MeOH vapor exposure.
Fig. S26 ^{1}H NMR spectra of a model complex, [$PtCl_2$(Si_2bpy)] before and after MeOH vapour exposure.
Fig. S27 ESI-MS (+) spectra of [Pt(ppy)(bpy)]Cl in pyridine.
Fig. S28 Preparation and vapour response of **PtCl+PMO** and **$PtPF_6$+PMO**.

Table S1 The XPS binding energies and the full-width at half-maxima.
Table S2 The BET surface area, pore volume, and average pore diameters.
Table S3 Photophysical data of complexes.
Table S4 Crystal parameters and refinement data of [Pt(ppy)(bpy)](PF_6).
Table S5 Selected interatomic distances and angles for [Pt(ppy)(bpy)](PF_6).

References

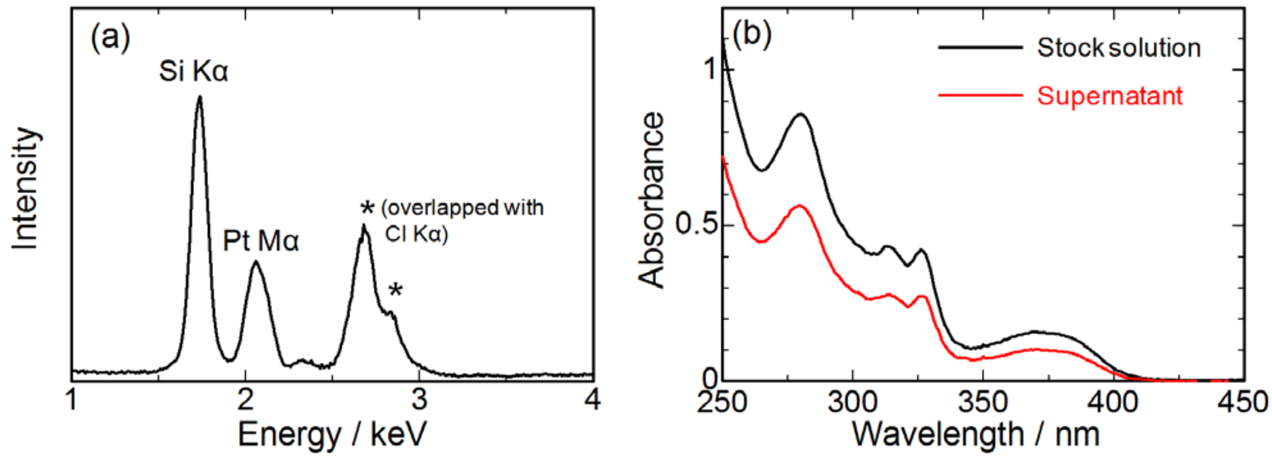

Fig. S1 (a) X-ray fluorescent (XRF) spectrum of **Pt-PMO**. Immobilized ratio was estimated from the ratio of the intensities of Si Kα and Pt Mα peaks by using the calibration curve. The peak marked by an asterisk originates from the Rh Lα and Lβ radiation of the X-ray source. (b) UV-vis absorption spectra of the stock solution (black) and supernatant solution of [Pt(ppy)(DMSO)Cl] after the reaction with BPy-PMO (red). These solutions were diluted 50 times by the solvent of each reaction. Immobilised ratio of Pt complex (i.e., reacted amount of [Pt(ppy)(DMSO)Cl]) was calculated by the absorbance at 370 nm (ε_{370} = 3.2 $\times 10^3$ $M^{-1}cm^{-1}$). From these two measurements, immobilised ratio is estimated to be 12 % Pt/Si.

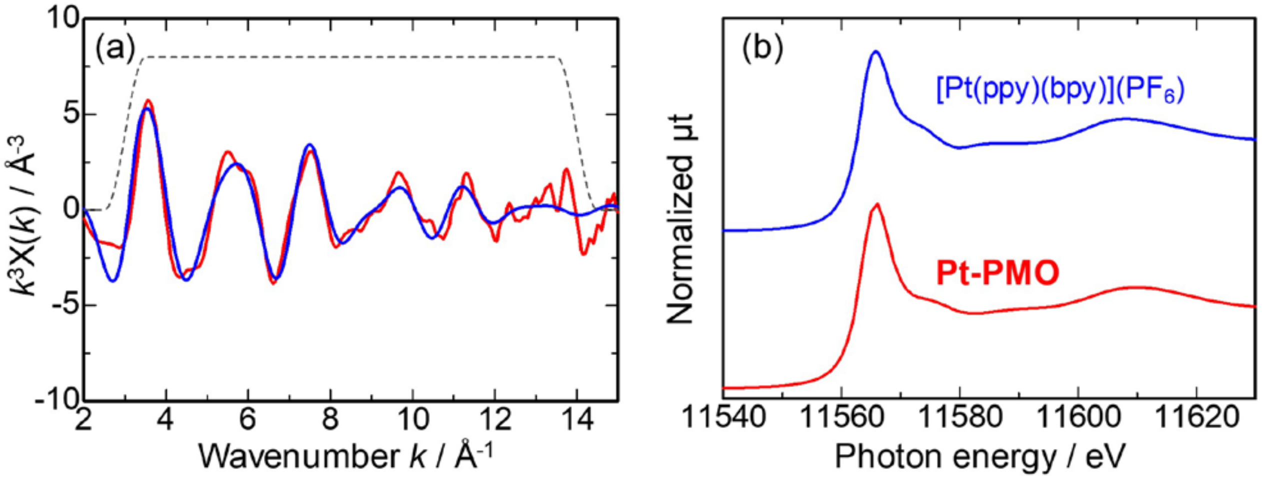

Fig. S2 (a) The k^3-weighted EXAFS spectrum of Pt-L_{III} edge of **Pt-PMO**. The experimental data, the fitting curve, and the Hanning window are shown in the red, blue, and broken gray lines, respectively. A fitting analysis of the EXAFS data was conducted based on the analyzed crystal structure of [Pt(bpy)(ppy)](PF_6) (Fig. S3). The bond lengths obtained by the fitting analysis were Pt-C: 2.01(1) Å (×1), Pt-N: 2.01(1) Å (×1), Pt-N: 2.05(1) Å (×2) for the 1st shell, and Pt-C: 2.63(3) Å (×2), Pt-C: 2.89(1) Å (×6) for the 2nd shell (Fig. 2b). The fitting parameters are as follows: Debye-Waller factor of the 1st shell, 0.00078(14) $Å^2$; Debye-Waller factor of the 2nd shell, 0.00097(24) $Å^2$; R-factor, 0.0138. (b) The XANES spectrum of Pt-L_{III} edge of **Pt-PMO** (red line) and [Pt(ppy)(bpy)](PF_6) (blue line).

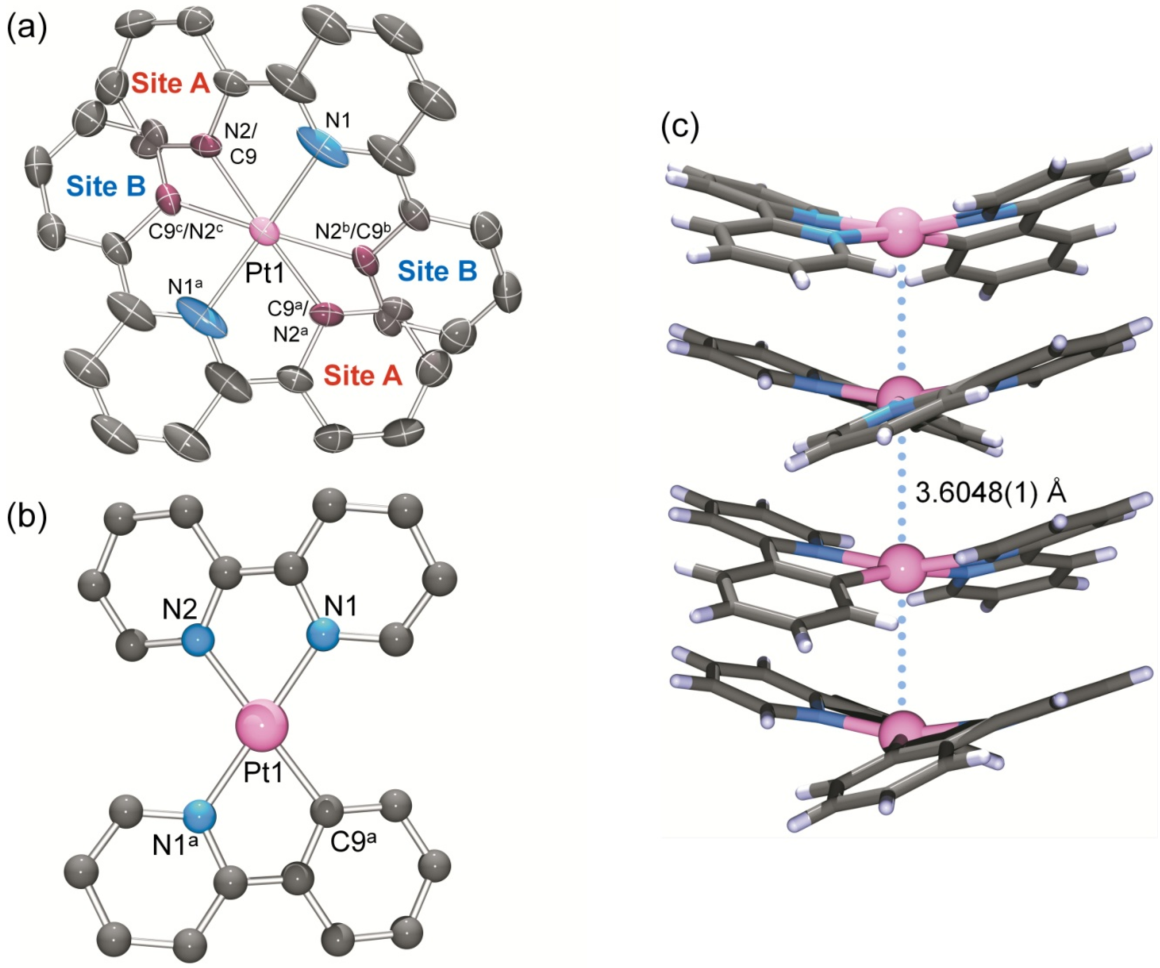

Fig. S3 (a) Crystal structure of the cationic part of [Pt(ppy)(bpy)](PF_6), showing the atom-labelling scheme. Thermal ellipsoids are displayed at the 50% probability level. Hydrogen atoms are omitted for clarity. (b) One of the two disordered structures of $[Pt(ppy)(bpy)]^+$. Hydrogen atoms are omitted for clarity. (c) Stacked arrangement of $[Pt(ppy)(bpy)]^+$.

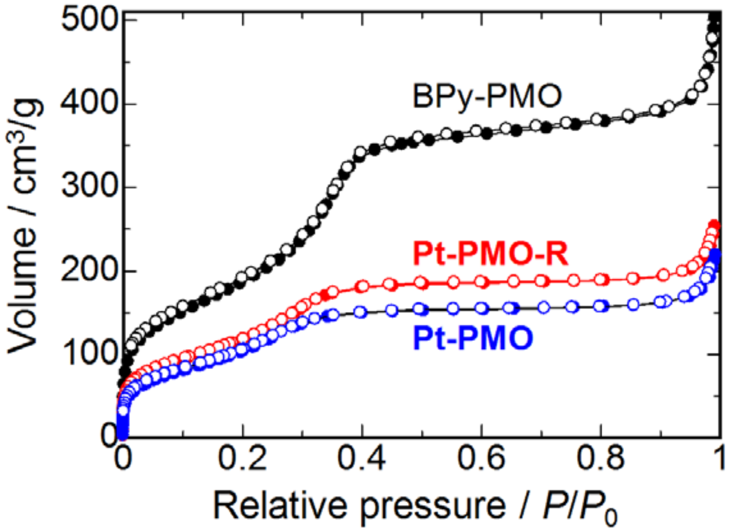

Fig. S4 Nitrogen adsorption desorption isotherm of BPy-PMO (black line), **Pt-PMO** (blue line) and **Pt-PMO-R** (red line) at 77 K. The Brunauer-Emmett-Teller (BET) surface area, all pore volume and average pore diameters are shown in Table S2.

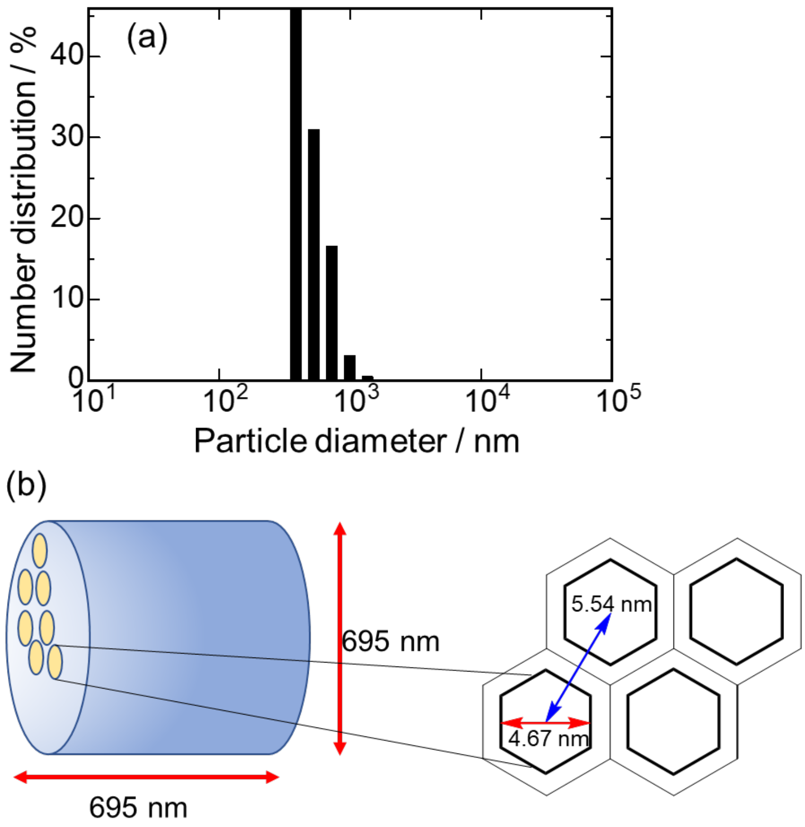

Fig. S5 (a) Particle diameter distributions estimated by the dynamic light scattering (DLS) of BPy-PMO, showing the average particle diameter of 695 nm. (b) Schematic illustration of the particle of BPy-PMO with a diameter of 695 nm based on the DLS, the average pore diameter of 4.7 nm based on the nitrogen adsorption isotherm, and the hexagonal lattice with a_0 = 5.54 nm based on the PXRD pattern. From these results, the ratio of outer to inner surface areas is estimated as follows, assuming cylindrical shapes of the particle and pores:

Number of pores = cylinder area / pore area = $(695/2)^2 \pi / (5.54/2)^2 \pi = 1.57 \times 10^4$

Ratio of inner to outer surface areas = pore surface areas / cylindrical particle surface area = $(4.67 \pi \times 1.57 \times 10^4) / 695\pi = 105$

The value means 99 % of surface is inside of BPy-PMO.

For the **Pt-PMO** sample with 12% Pt/Si, the abundance ratio of the bpy groups in BPy-PMO is roughly estimated as follows:

outer : middle : inner = 0.095 : 0.59 : 1 = 0.006 : 0.37 : 0.63,

where the ratio of the middle layer is estimated to be (5.54/2)/4.67 = 0.59

For the **Pt-PMO** sample with 12% Pt/Si, the ratio of Pt/bpy is 24 %. Therefore, approximately 37 % (i.e. 0.234/0.63) of the bpy moieties on the inner surface are estimated to be occupied by the Pt-ppy units, assuming a full occupation of the outer surface bpy moieties by the Pt complexes,

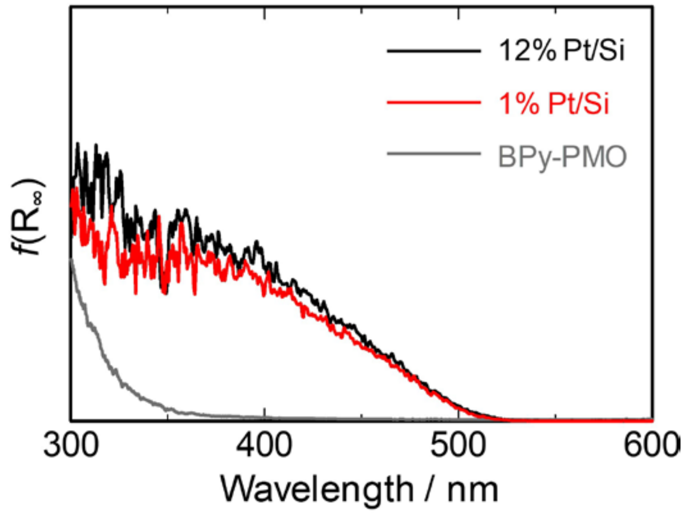

Fig. S6 UV-vis diffuse reflectance spectra of **Pt-PMO** with the immobilised ratio of 12% Pt/Si (black lines) and 1% Pt/Si (red lines). Grey line shows the UV-vis diffuse reflectance spectrum of BPy-PMO.

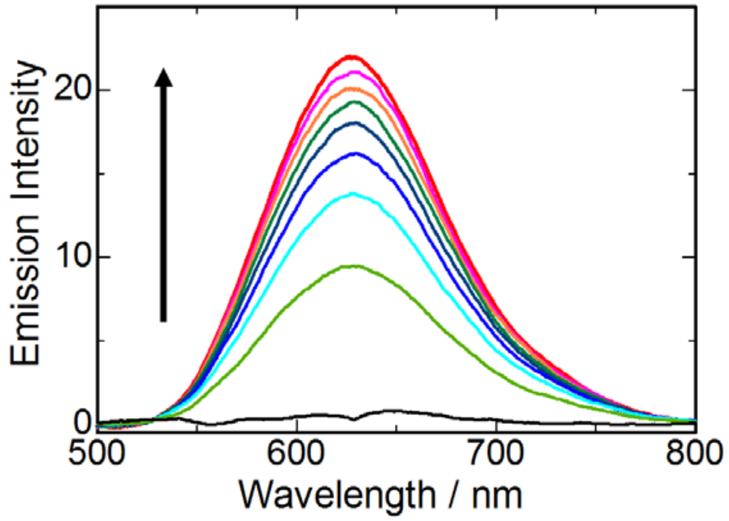

Fig. S7. Evolution of the emission spectrum of **Pt-PMO** during MeOH vapour exposure (from **Pt-PMO** to **Pt-PMO-R**) at 50°C (λ_{ex} = 410 nm). Spectra were recorded at 1-h intervals.

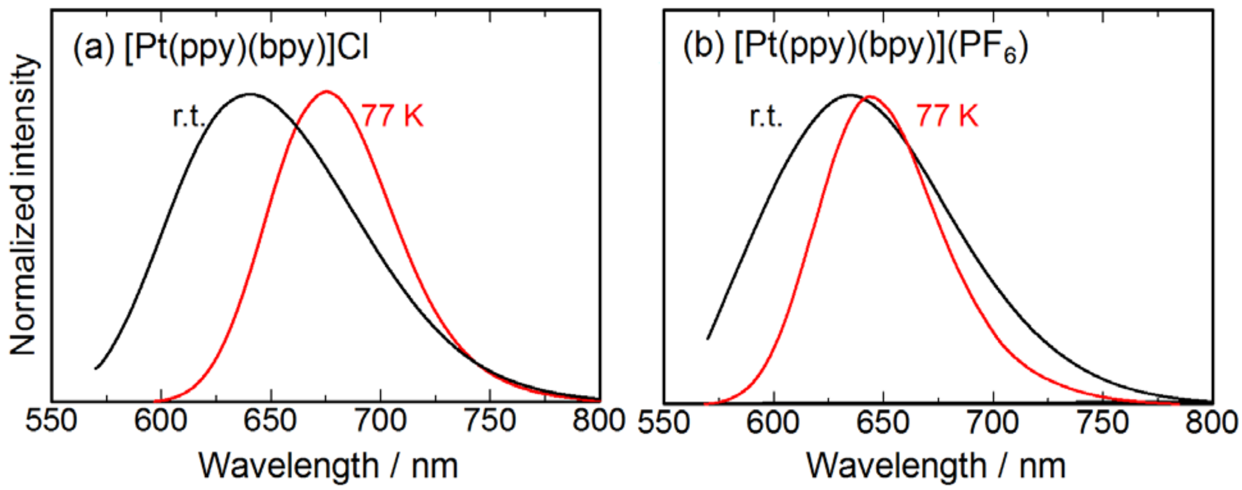

Fig. S8 Emission spectra of (a) [Pt(ppy)(bpy)]Cl (b) [Pt(ppy)(bpy)](PF_6) in the solid state at r.t. (black line) and 77 K (red line) (λ_{ex} = 540 nm). At 77 K, the emission bands showed some red-shift as typically observed for the ^{3}MMLCT emission of Pt(II) complexes with Pt···Pt interaction.[1] The photophysical data are summarised in Table S3.

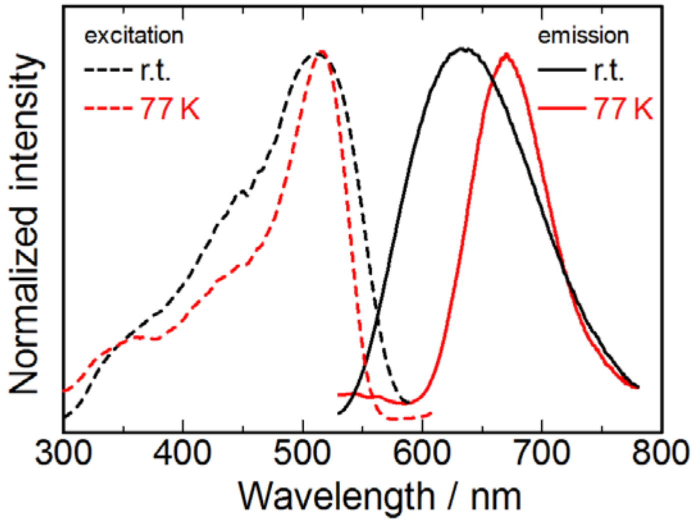

Fig. S9 Emission (solid lines) and excitation (broken lines) spectra of **Pt-PMO-R** at r.t. (black lines) and 77 K (red lines). The excitation wavelengths for the emission spectra were 535 and 532 nm for r.t. and 77 K, respectively. The detection wavelengths for the excitation spectra were 630 and 673 nm for r.t. and 77 K, respectively.

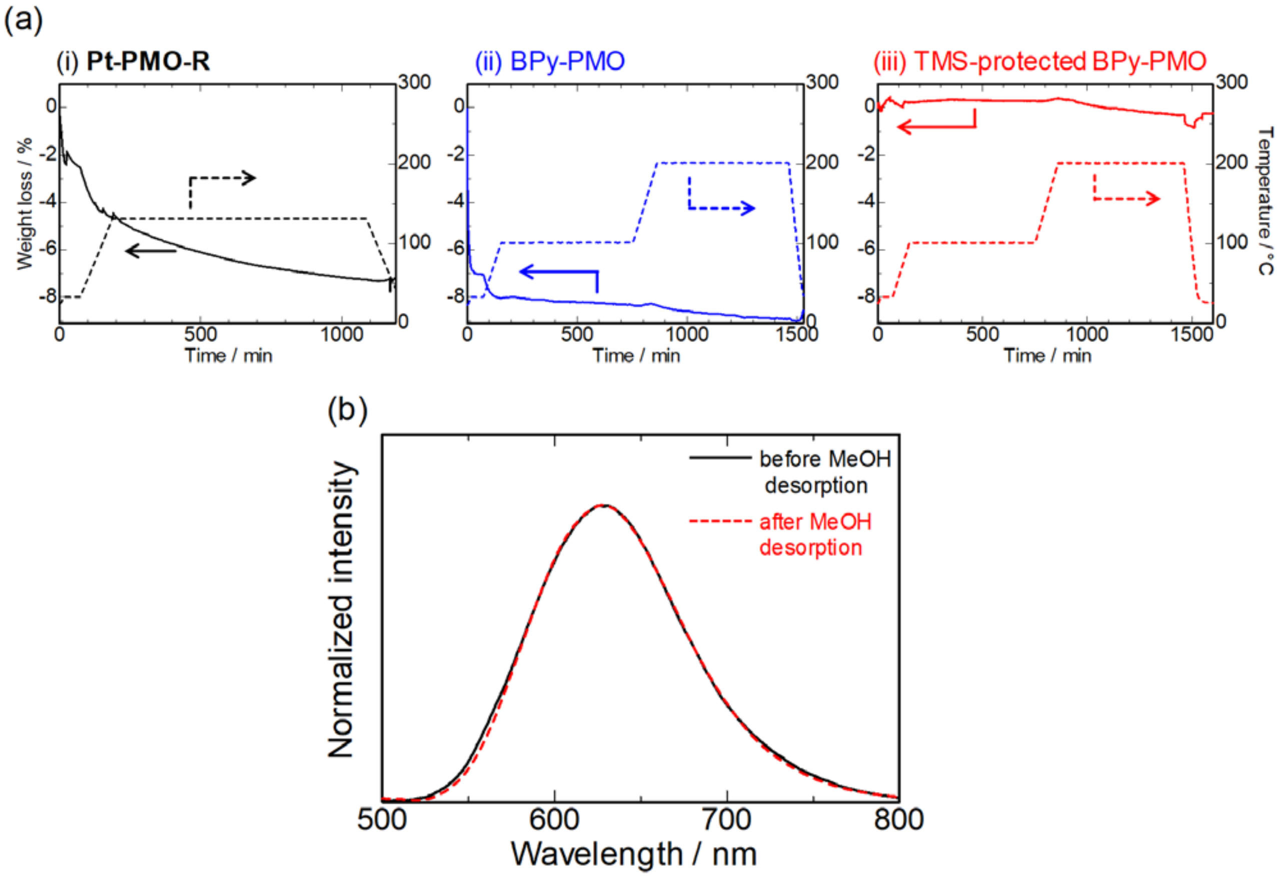

Fig. S10 (a) Thermogravimetric analysis for **Pt-PMO-R** (i), BPy-PMO (ii), and trimethylsilyl (TMS)-protected BPy-PMO (iii) after the exposure to methanol vapour at 323 K. For **Pt-PMO-R** and BPy-PMO, 7.3 wt% and 9.0 wt% of weight loss, corresponding to the desorption of methanol (0.9 mol/mol based on bpy unit), were observed. On the other hand, no weight loss was observed after the protection of silanol by trimethylsilyl groups. (b) emission spectra of **Pt-PMO-R** before (black line) and after (broken red line) the desorption of methanol by heating at 403 K. As a result, almost no change was observed after heating, indicating the high stability of the methanol-detected form **Pt-PMO-R**.

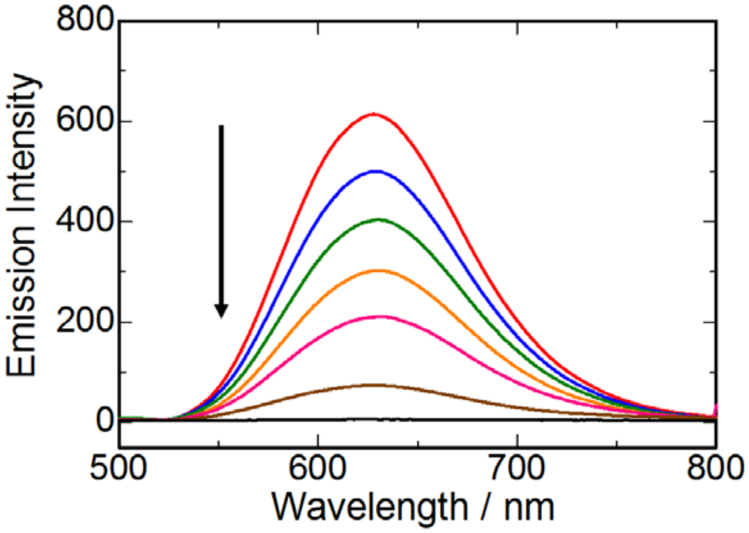

(a)

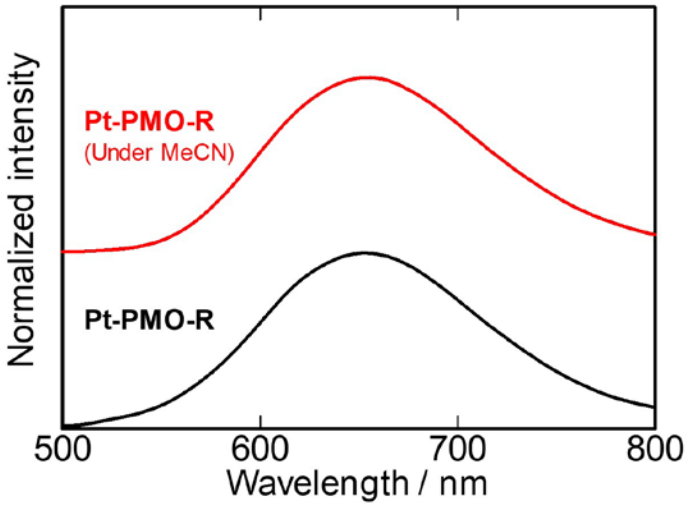

(b)

Fig. S11 (a) Changes in the emission spectrum of **Pt-PMO-R** during pyridine vapour exposure (from **Pt-PMO-R** to **Pt-PMO-LY**) at 50°C (λ_{ex} = 410 nm). Spectra were recorded at 5-min intervals. (b) Emission spectra of **Pt-PMO-R** before (black) and after the exposure to acetonitrile (MeCN) vapour (red) for 1 h at 50 °C (λ_{ex} = 450 nm).

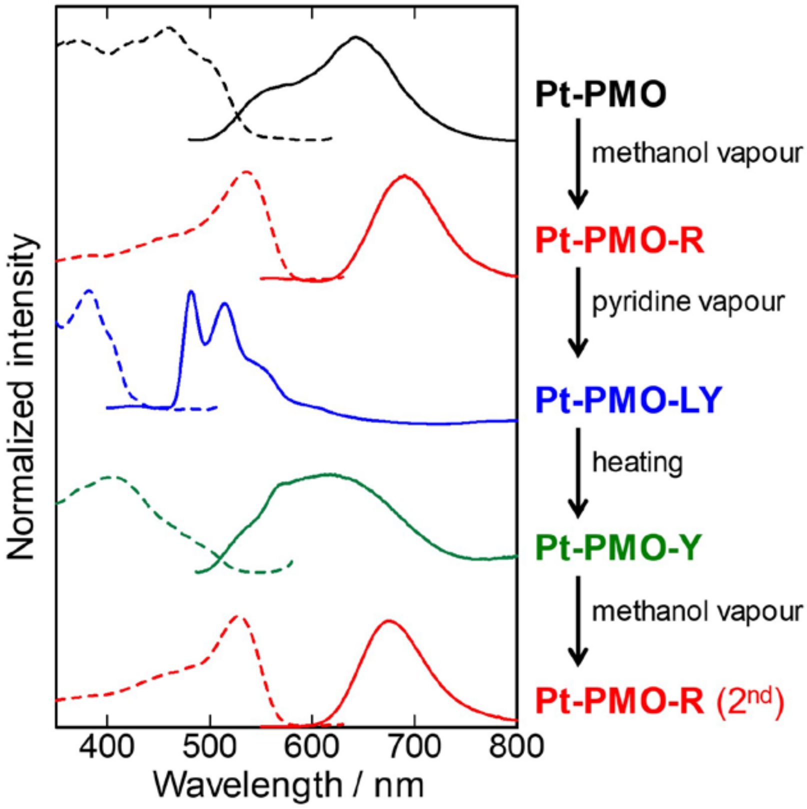

Fig. S12 Emission (solid lines) and excitation (broken lines) spectral change of **Pt-PMO** at 77 K during the vapour response cycle. Both **Pt-PMO-R** (1st) and **Pt-PMO-R** (2nd) showed almost identical emission spectra, suggesting the chemical compositions of them are same. In addition, after the pyridine vapour exposure, an emission spectrum of **Pt-PMO-LY** clearly showed the vibrational progression assignable to the $^3\pi\pi^*$ emission. This emission band of **Pt-PMO-LY** is almost identical to that of [Pt(ppy)(bpy)]Cl in solution (Fig. 3), indicating negligible Pt···Pt interactions in this form. Excitation wavelengths were 450 nm for **Pt-PMO-R** and 410 nm for other samples.

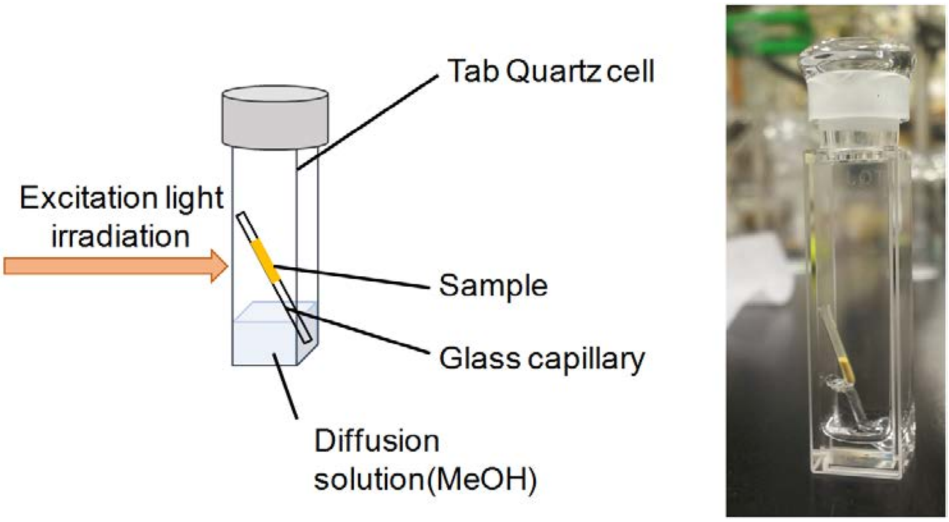

Fig. S13 Schematic image and the photograph of the experimental setup for the measurement of emission spectral changes of samples under vapour exposure. A sample was put in a glass capillary whose top was opened for the vapour diffusion. Then the capillary was placed in a quartz cell which was warmed with a Peltier device at 50 °C. A small amount of methanol was introduced into the quartz cell, and the measurements was started immediately. As shown in Fig. 5a, some induction period was needed for the vapor diffusion into the glass capillary.

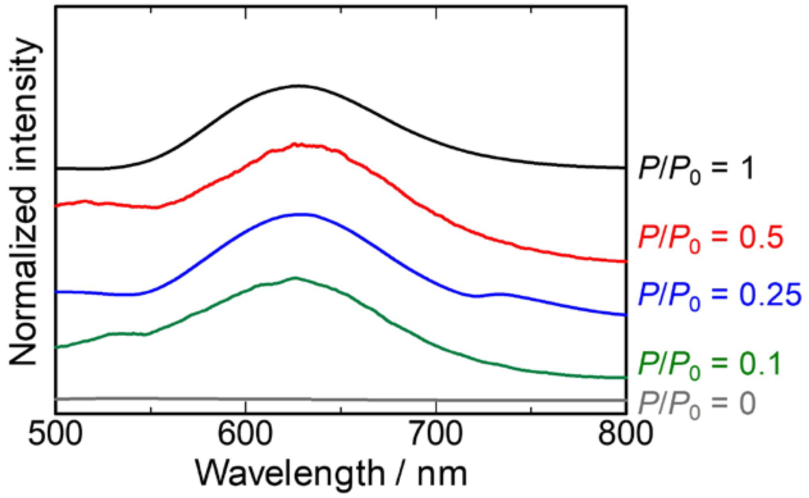

Fig. S14 Emission spectra of **Pt-PMO-Y** after the exposure to various relative pressures of methanol vapour (λ_{ex} = 410 nm). **Pt-PMO-Y** showed vapochromic luminescence even at P/P_0 = 0.1 although relatively long exposure time was required (up to 1 h).

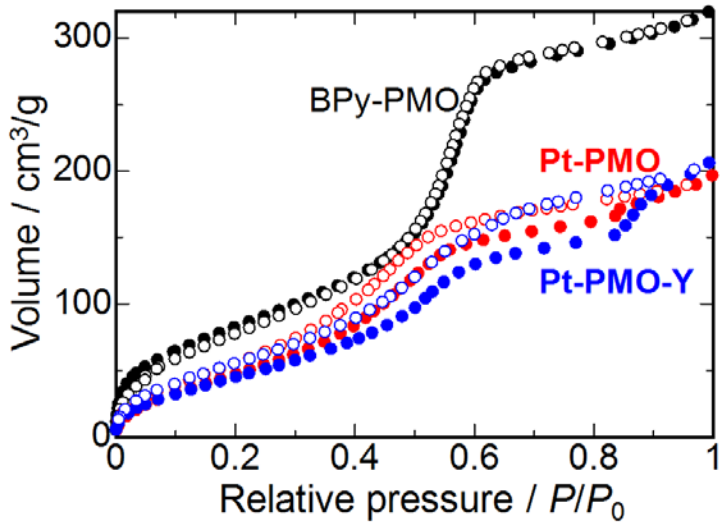

Fig. S15 Methanol vapour adsorption isotherms of BPy-PMO (black), **Pt-PMO** (red), **Pt-PMO-Y** (blue) at 298 K.

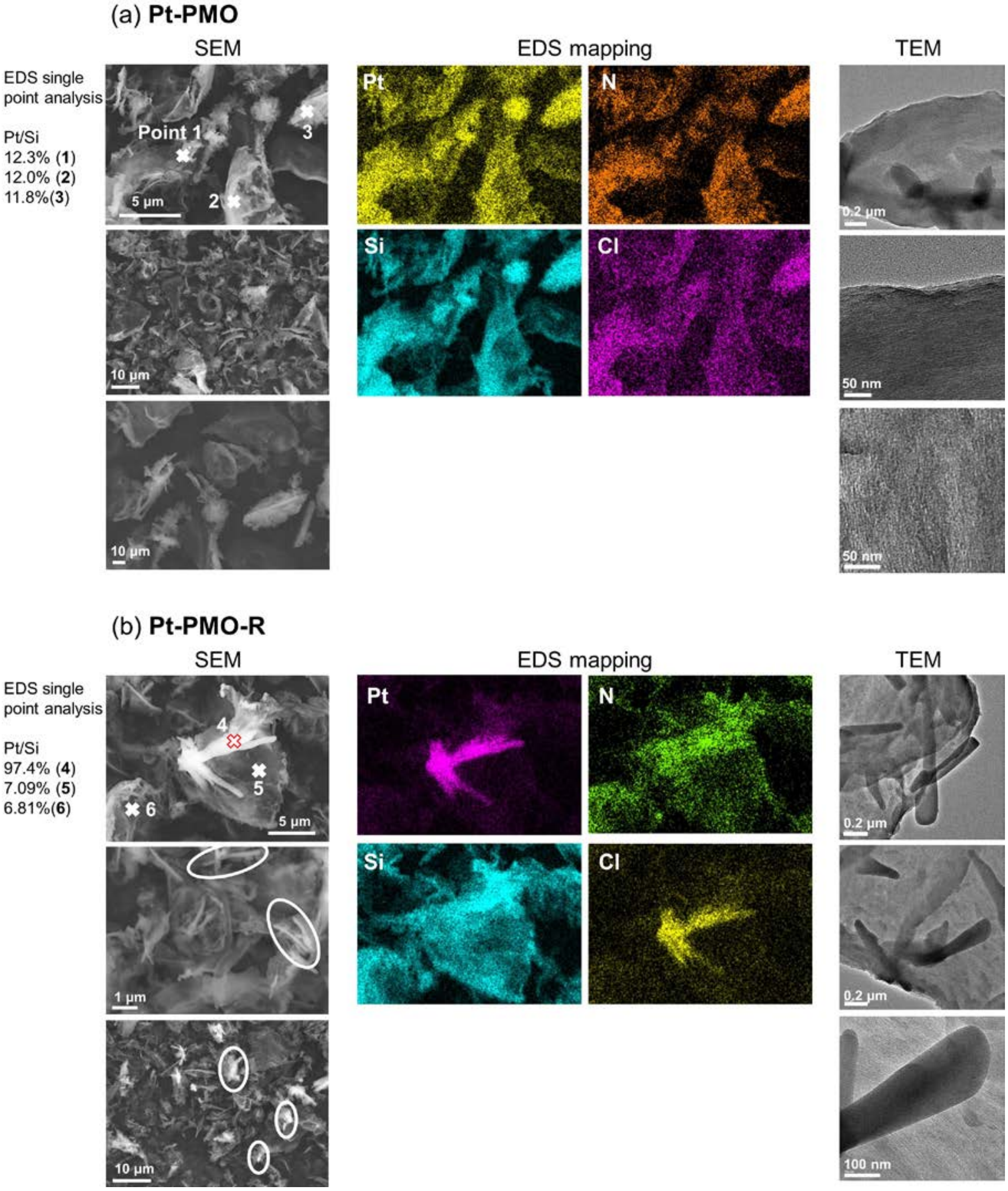

Fig. S16 SEM images, EDS elemental mapping at Pt M-edge, Si K-edge, N K-edge, and Cl K-edge, and TEM images of (a) **Pt-PMO**, (b) **Pt-PMO-R**, (c) **Pt-PMO-LY**, (d) **Pt-PMO-Y**, and (e) **Pt-PMO-R** (2nd exposure).

(c) **Pt-PMO-LY**

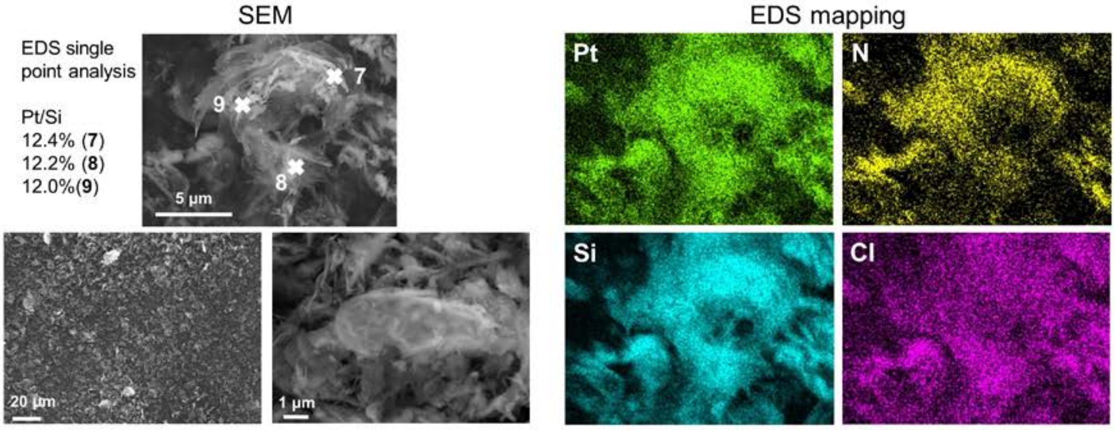

(d) **Pt-PMO-Y**

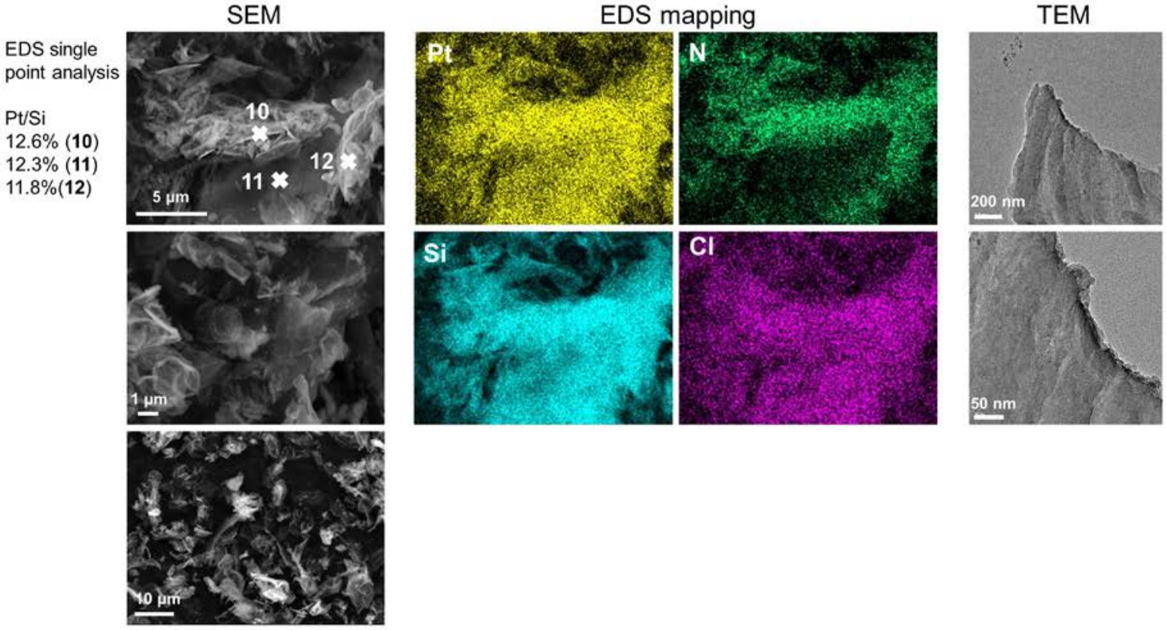

(e) **Pt-PMO-R** (2nd)

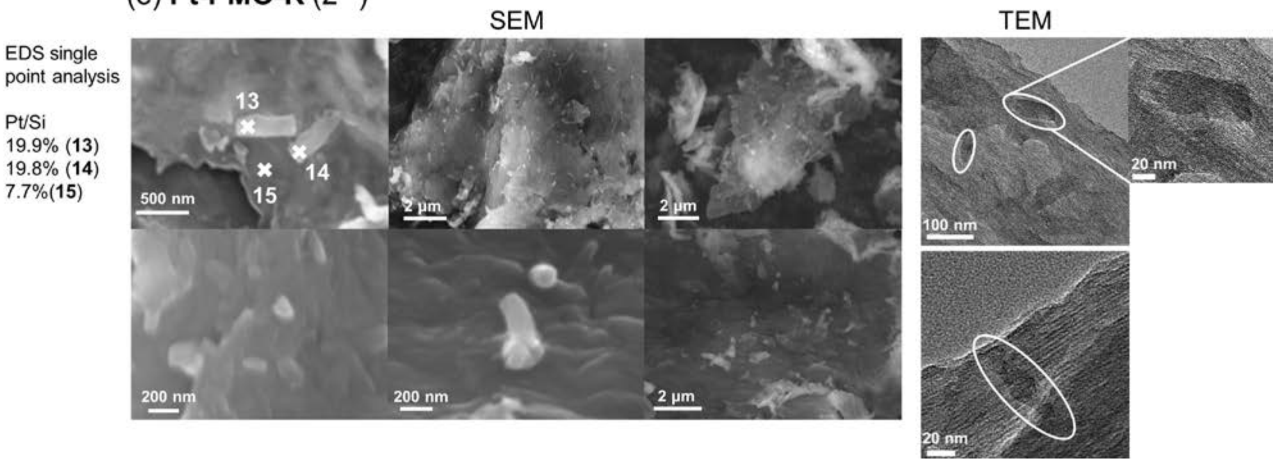

Fig. S16 (Continued)

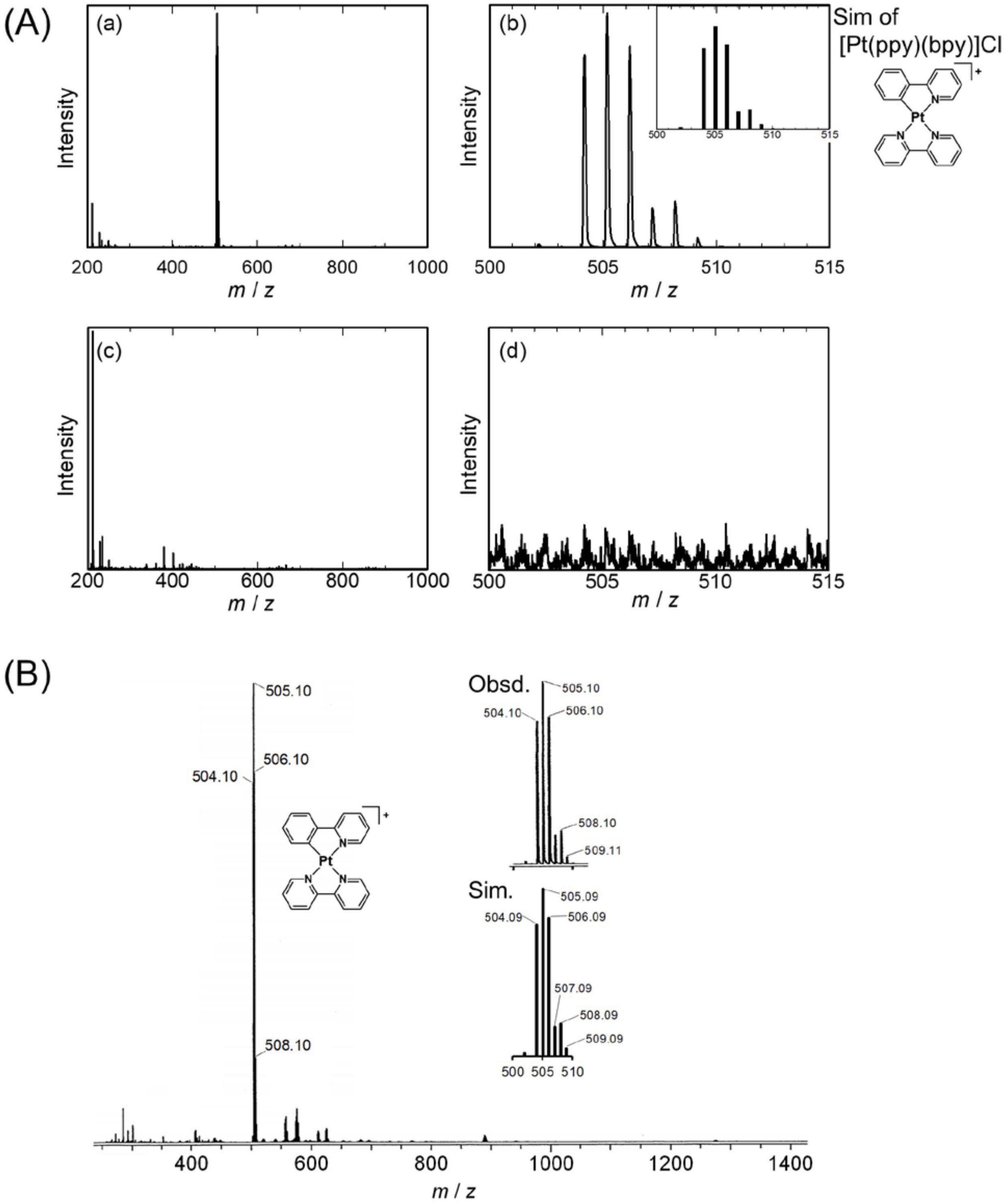

Fig. S17 (A) MALDI-MS of powder samples of (a,b) **Pt-PMO-R** and (c,d) **Pt-PMO** (positive mode, matrix: CHCA), where (b) and (d) are the expansion of the *m/z* = 500–515 region. (B) ESI-MS of the soluble species of **Pt-PMO-R** (positive mode, methanol). Mass spectrum of **Pt-PMO-R** clearly showed the main signal at *m/z* = 505.1 corresponding to $[Pt(ppy)(bpy)]^+$, while such signal was not observed before the methanol vapour exposure (**Pt-PMO**).

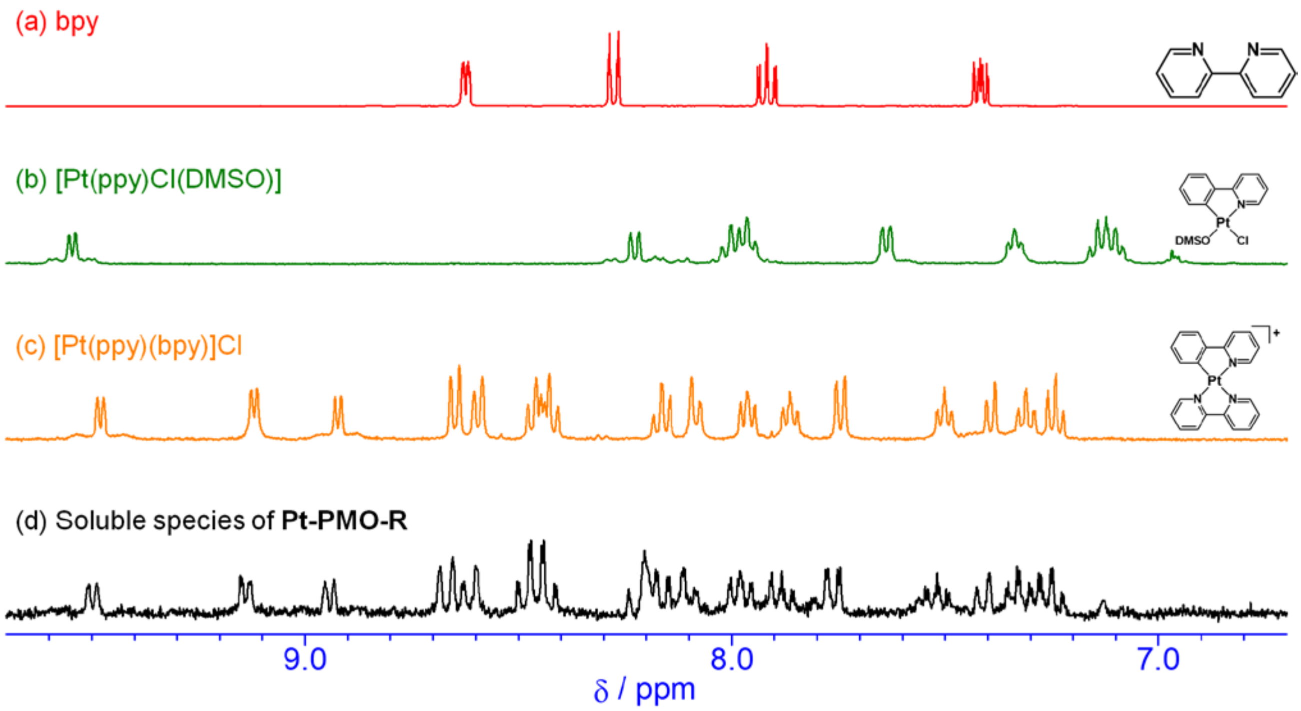

Fig. S18 ^{1}H NMR spectra (methanol-d_4) of (a) bpy (400 MHz), (b) [Pt(ppy)Cl(DMSO)] (400 MHz), (c) [Pt(ppy)(bpy)]Cl (400 MHz), and (d) the soluble species of **Pt-PMO-R** (270 MHz). The spectrum of the soluble species of **Pt-PMO-R** is consistent with that of [Pt(ppy)(bpy)]Cl, and different from those of bpy and [Pt(ppy)Cl(DMSO)].

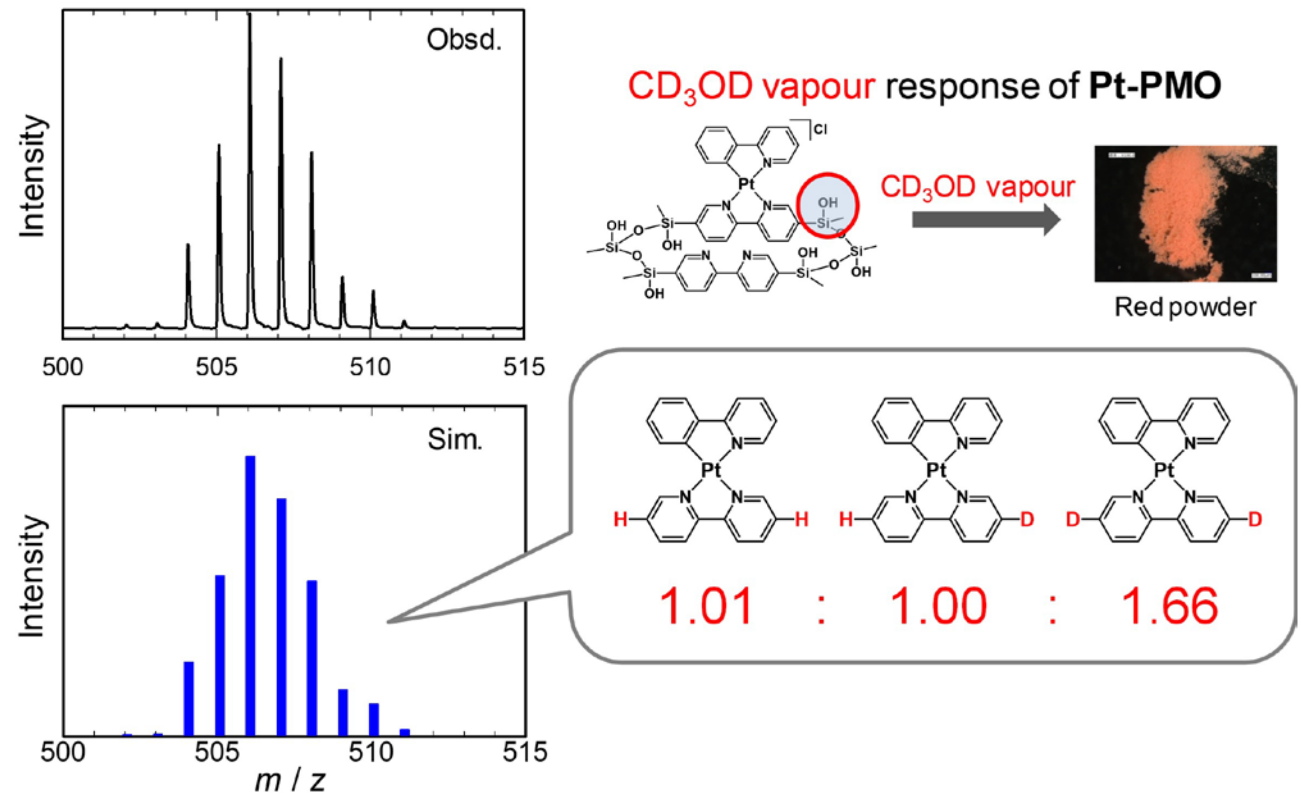

Fig. S19 ESI-MS of the soluble species of **Pt-PMO** after the methanol-d_4 vapour exposure (top; hereafter termed as **Pt-PMO-R(d)**). Observed spectrum was clearly different from that of **Pt-PMO-R**. Actually, the isotope pattern of the observed spectrum was reproduced by assuming the presence of non-, mono-, and dideuterated [Pt(ppy)(bpy)Cl]$^+$ in the ratio of 1.01: 1.00: 1.66 (bottom; H:D = 1.00: 1.43; in other words, 59% of 5,5′-protons were deuterated), indicating the incorporation of protons of methanol vapour as well as the silanol groups on PMO.

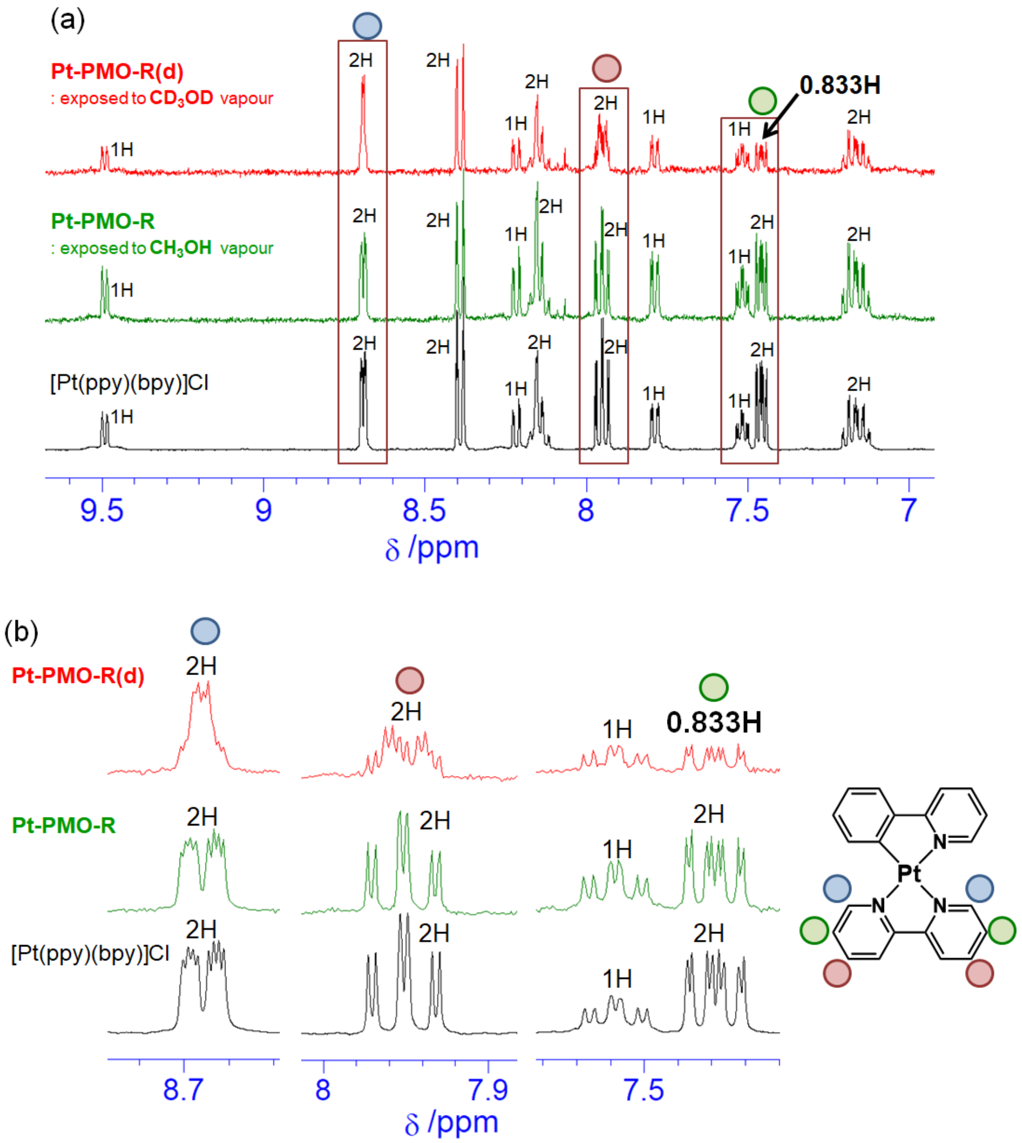

Fig. S20 (a) ^{1}H NMR spectra (400 MHz, DMSO-d_6) of the soluble species of **Pt-PMO-R(d)** (red lines) and **Pt-PMO-R** (green lines), which were prepared by using methanol-d_4 or methanol vapour, respectively. Black lines show the ^{1}H NMR spectrum of [Pt(ppy)(bpy)]Cl. (b) The expansion around the 4,4′-, 5,5′-, and 6,6′-protons of bpy. By using methanol-d_4 (**Pt-PMO-R(d)**), the intensity of signal corresponding to 5,5′-protons of bpy (indicated by green markers) decreased from 2.0 to 0.833, indicating ca. 58% deuteration of 5,5′-protons. This value is consistent with the ESI-MS result (Fig. S19). In addition, the splitting of signals corresponding to 4,4′- and 6,6′-protons of bpy, indicated by pink and blue markers respectively, largely changed due to the partial deuteration of 5,5′-protons. These protons were assigned from the ^{1}H,^{1}H-COSY NMR spectrum of [Pt(ppy)(bpy)]Cl (400 MHz, DMSO-d_6) shown in (c). Although [Pt(ppy)(bpy)]Cl was found to be decomposed to [Pt(ppy)(DMSO-d_6)Cl] and bpy in DMSO-d_6, (see the reported chemical shifts of [Pt(ppy)(DMSO)Cl][3] and bpy[4]), we have selected DMSO-d_6 instead of methanol-d_4 to avoid the protodesilylation during the extraction of the soluble species.

(c)

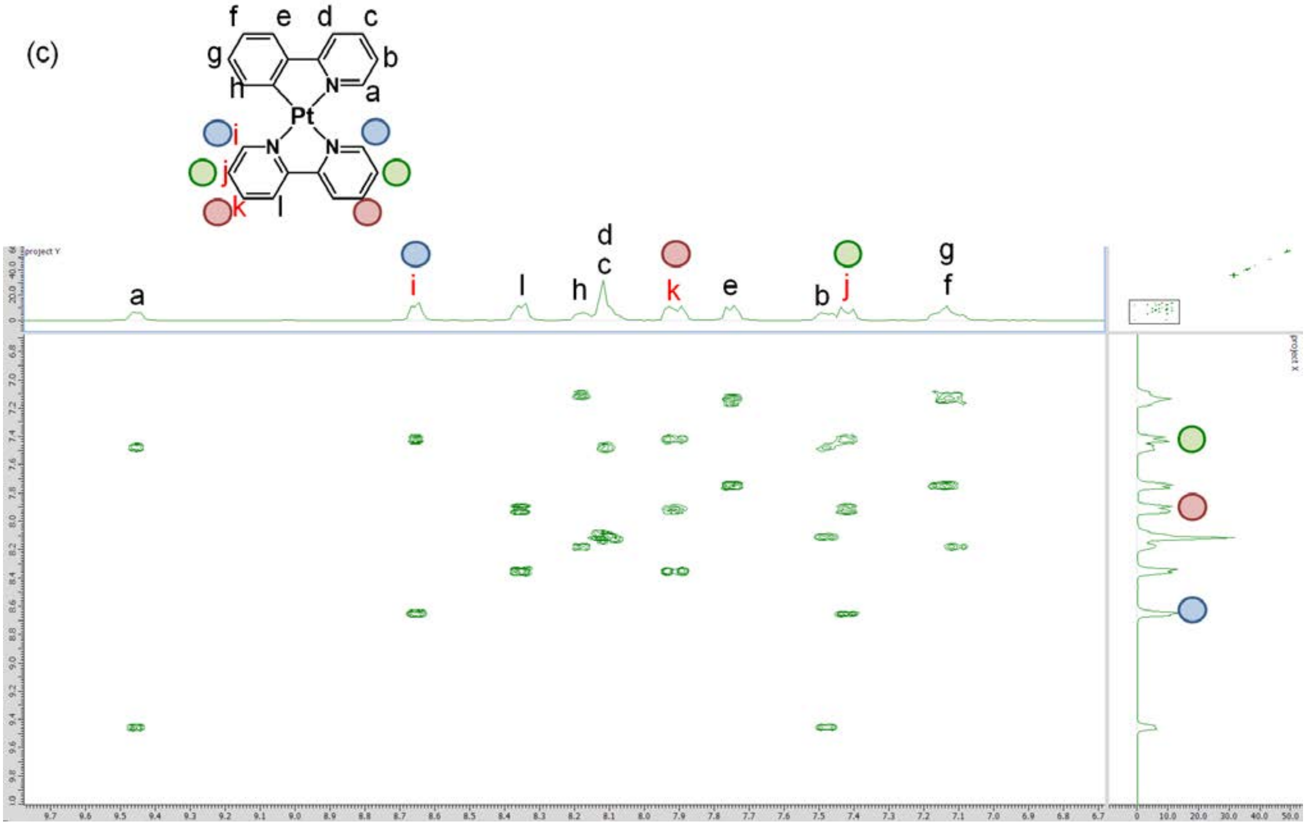

Fig. S20 (continued)

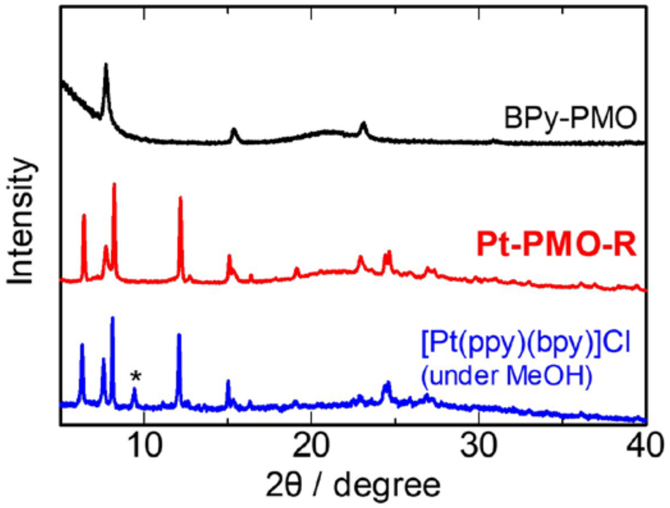

Fig. S21 PXRD patterns of BPy-PMO (black line), **Pt-PMO-R** (red line) and [Pt(ppy)(bpy)]Cl under MeOH vapor (blue line). The peak marked with an asterisk would originate from the desolvated form of [Pt(ppy)(bpy)]Cl (see Fig. S24).

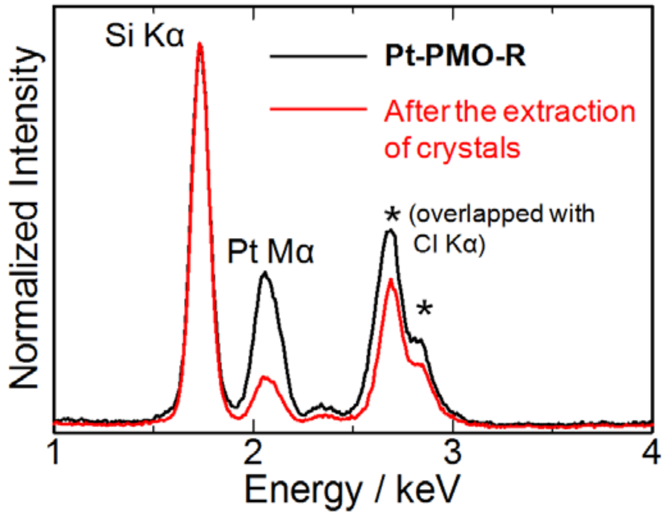

Fig. S22 X-ray fluorescent (XRF) spectrum of **Pt-PMO-R** before (black line) and after (red line) the extraction of the crystalline [Pt(ppy)(bpy)]Cl. The peak marked by an asterisk originates from the Rh Lα and Lβ radiation of the X-ray source. Intensities were normalised at the Si Kα peak, and immobilized ratios were estimated from the ratio of the intensities of Si Kα and Pt Mα peaks. As a result, that the immobilised ratio was largely decreased from 12% to 4% Pt/Si. So the 2/3 of immobilised Pt(II) complex (8 % Pt/Si) was detached from BPy-PMO via the protodesilylation.

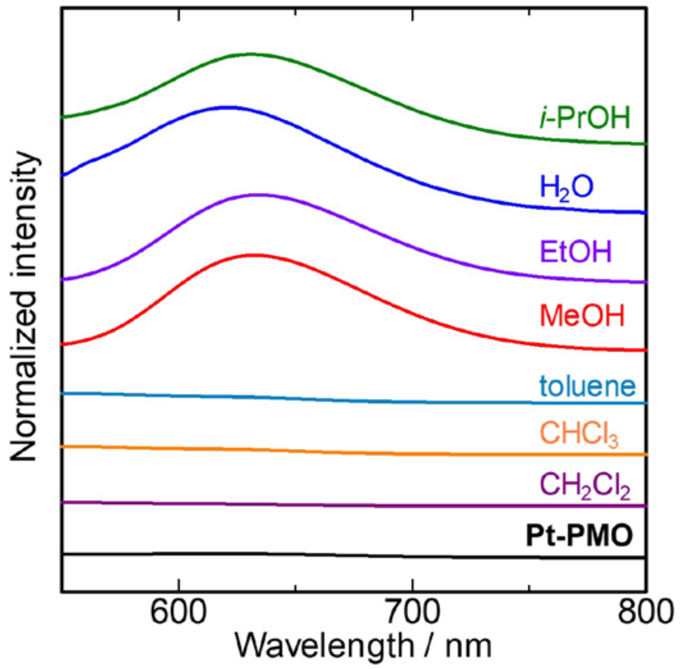

Fig. S23 Emission spectra of **Pt-PMO** before (black) and after the exposure to several vapour such as MeOH (red), EtOH (purple), H_2O (blue), *i*-PrOH (green), CH_2Cl_2 (red-purple), $CHCl_3$ (orange), and toluene (light blue).

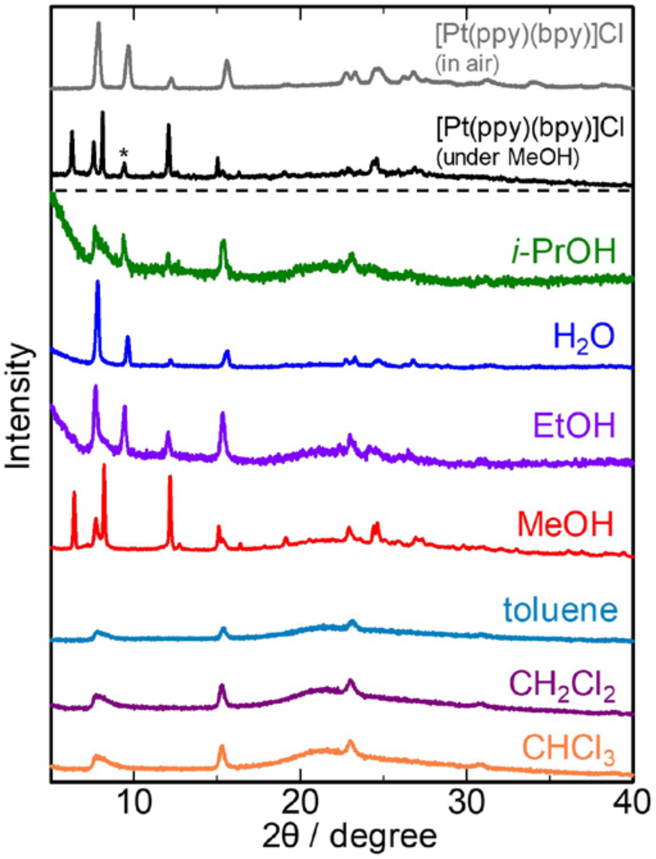

Fig. S24 PXRD patterns of **Pt-PMO** after exposure to several vapours such as MeOH (red), EtOH (purple), H_2O (blue), *i*-PrOH (green), CH_2Cl_2 (red-purple), $CHCl_3$ (orange) and toluene (light blue). Black and gray lines show the PXRD pattern of [Pt(ppy)(bpy)]Cl under MeOH vapor (black) or in air (gray). The peak marked by an asterisk, which is absent in the PXRD pattern of **Pt-PMO-R**, would originate from the desolvated form of [Pt(ppy)(bpy)]Cl (i.e., measured in air).

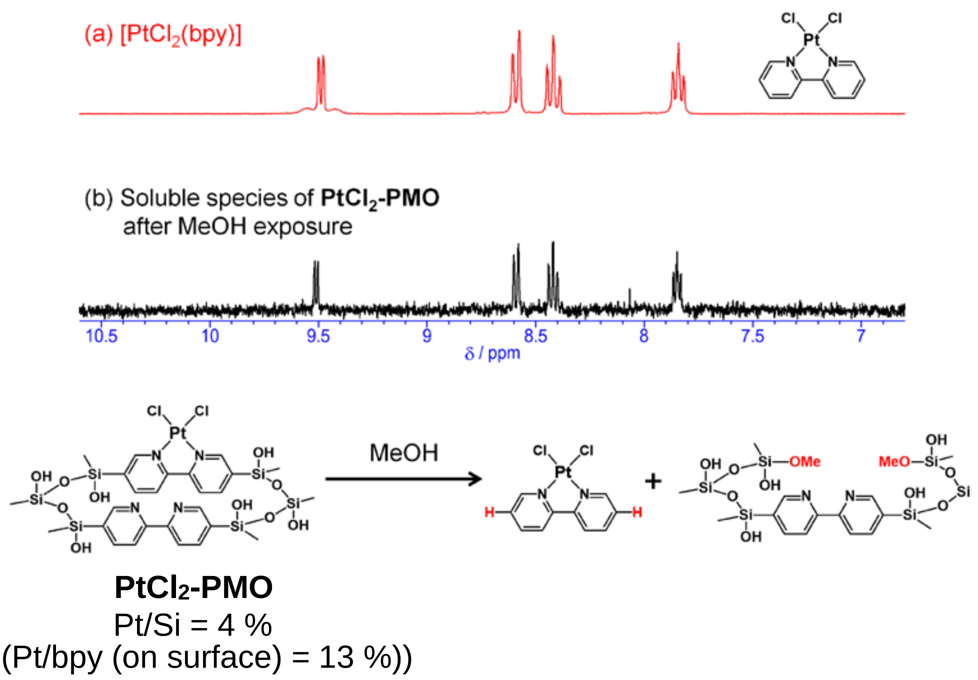

Fig. S25 ^{1}H NMR spectra (DMSO-d_6) of (a) [$PtCl_2$(bpy)] (270 MHz), and (b) the soluble species of **$PtCl_2$-PMO** after the MeOH vapor exposure (400 MHz). The spectrum of the soluble species after the exposure is consistent with that of [$PtCl_2$(bpy)], indicating that the methanol vapor-induced Si-C bond dissociation occurred also in the Pt-immobilized system.

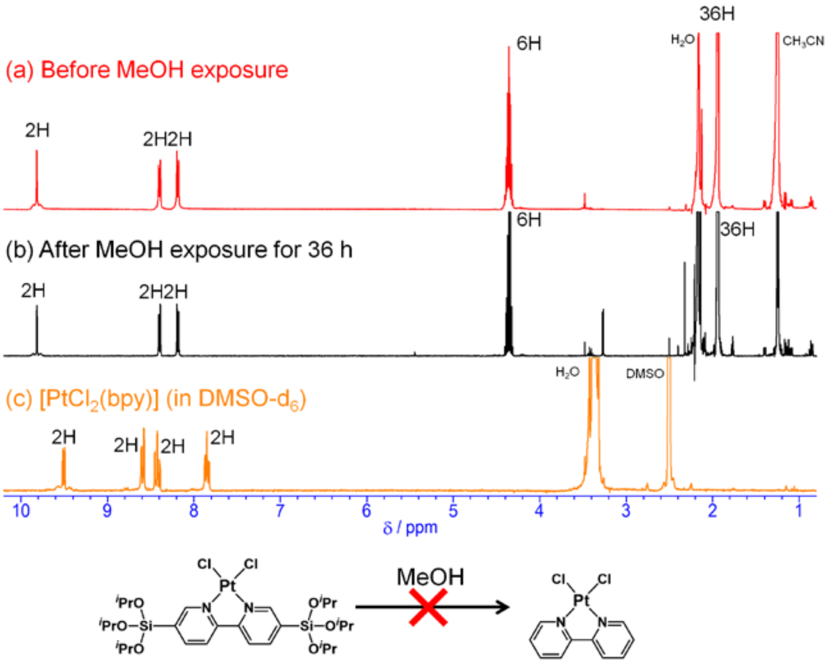

Fig. S26 ^{1}H NMR spectra of a model complex, [PtCl$_2$(Si$_2$bpy)] (400 MHz, CD$_3$CN) before (a) and after (b) MeOH vapor exposure for 36 h at 50 °C, and (c) [PtCl$_2$(bpy)] (270 MHz, DMSO-d$_6$). The protodesilylation did not proceed for [PtCl$_2$(Si$_2$bpy)], indicating the importance of the porous structure of **PtCl$_2$-PMO** for the reaction.

Synthesis of [PtCl$_2$(Si$_2$bpy)]. A solution of *cis*-[PtCl$_2$(DMSO)$_2$] (43.5 mg, 0.103 mmol) and Si$_2$bpy (57.9 mg, 0.103 mmol) in CHCl$_3$ (5 mL) was refluxed for 3 h. The resulting mixture was concentrated under reduced pressure followed by the addition of diethyl ether. The yellow precipitate was collected through filtration, washed with diethyl ether, and dried *in vacuo*. Yield: 73.5 mg (0.088 mmol, 86%). ^{1}H NMR (400 MHz, CD$_3$CN, δ): 9.79 (s, 2H), 8.37 (d, 2H, J = 7.9 Hz), 8.16 (d, 2H, J = 8.0 Hz), 4.33 (m, 6H), 1.22 (d, 36H, J = 6.0 Hz).

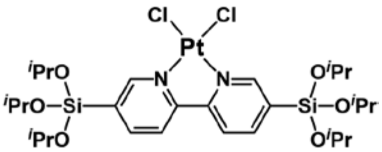

[PtCl$_2$(Si$_2$bpy)]

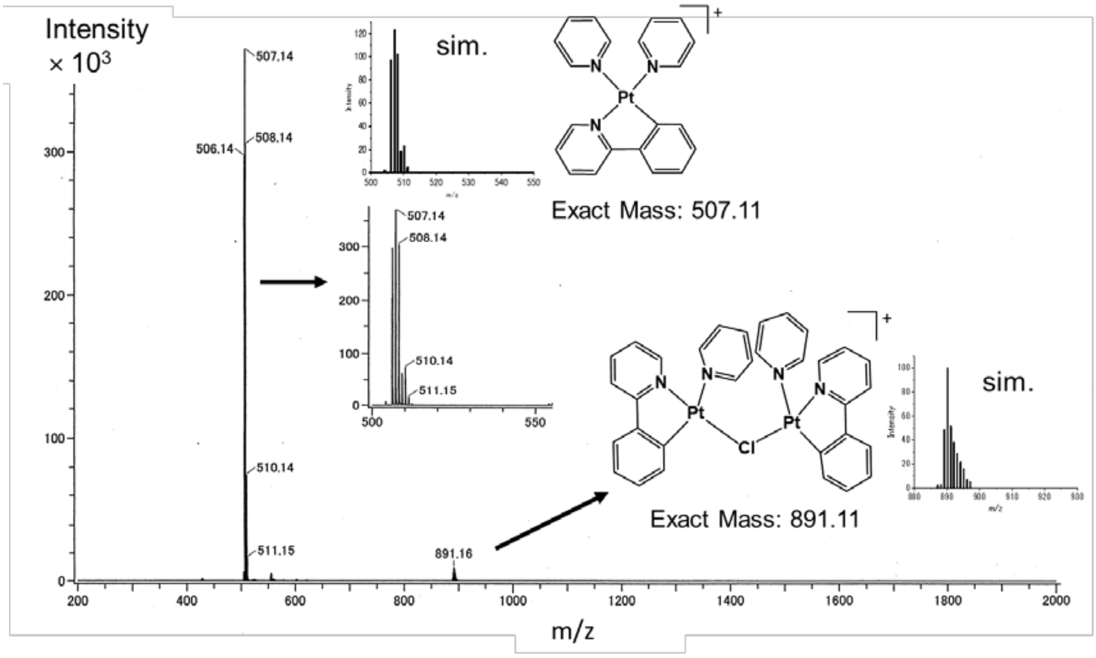

Fig. S27 ESI-MS (+) spectra of [Pt(ppy)(bpy)]Cl in pyridine.

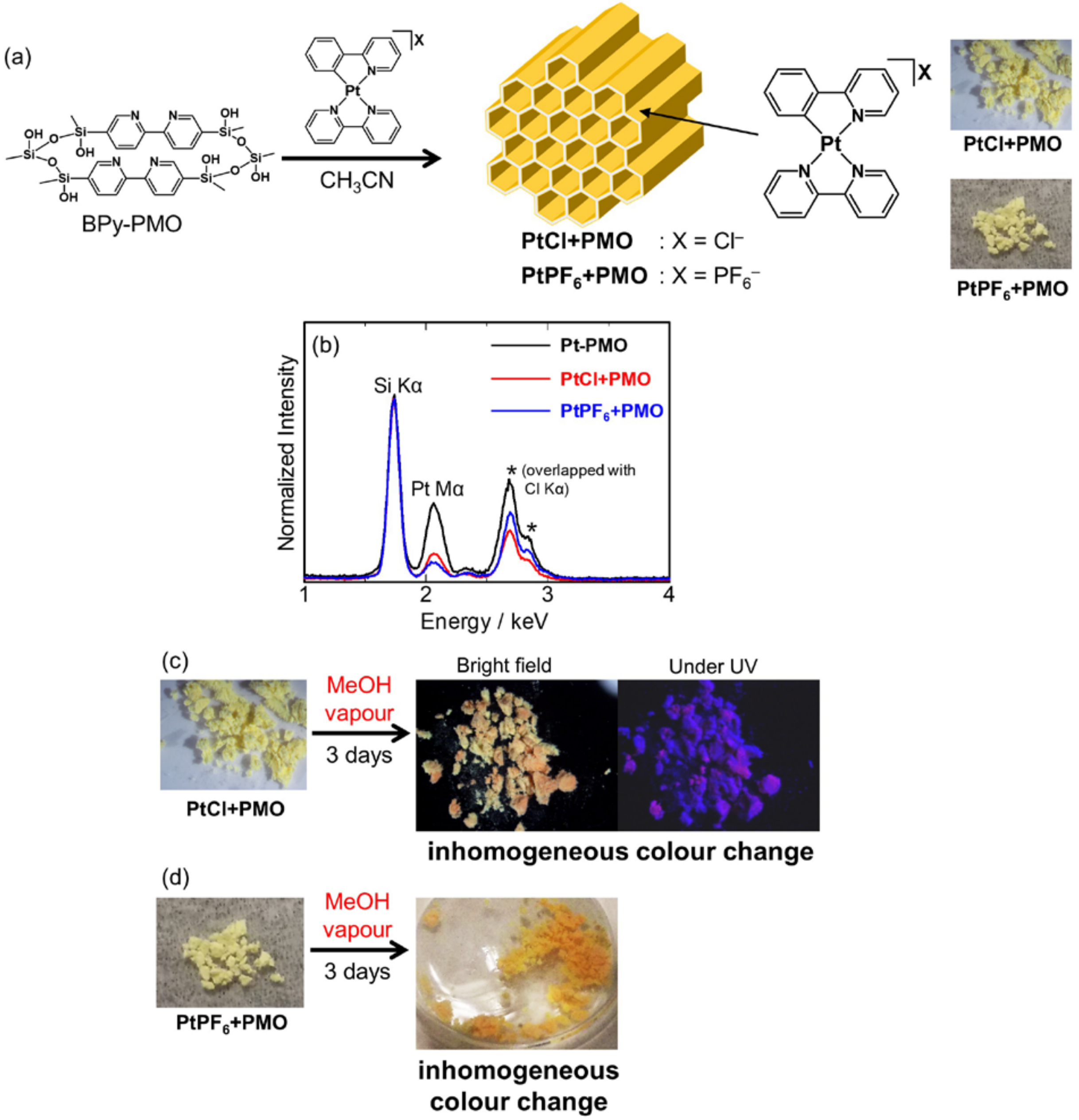

Fig. S28 (a-b) Preparation and (c,d) vapour response of **PtCl+PMO** and **PtPF$_6$+PMO**, where the Pt(II) complex was physisorbed onto BPy-PMO). (a) Synthetic scheme of **PtCl+PMO** and **PtPF$_6$+PMO**. The details of the synthetic method were described in the next page. (b) XRF spectra of **Pt-PMO** (black line), **PtCl+PMO** (red line), and **PtPF$_6$+PMO** (blue line). The peak marked by an asterisk originates from the Rh Lα and Lβ radiation of the X-ray source. Intensities were normalized at the Si Kα peak, and immobilized ratios were estimated from the ratio of the intensities of Si Kα and Pt Mα peaks. As a result, the immobilized ratio of **PtCl+PMO** and **PtPF$_6$+PMO** were estimated to be 3% and 2% Pt/Si. (c,d) Colour change of **PtCl+PMO** and **PtPF$_6$+PMO** under methanol vapour exposure for 3 days. Both **PtCl+PMO** and **PtPF$_6$+PMO** showed inhomogeneous colour change, and this colour change require longer vapour exposure time than that for **Pt-PMO** (8 h).

Synthesis of [Pt(ppy)(bpy)]Cl physisorbed on BPy-PMO (PtCl+PMO). A suspension of BPy-PMO (13.4 mg, 0.05 mmol) and [Pt(ppy)(bpy)]Cl (27.0 mg, 0.05 mmol) in CH_3CN (15 ml) was refluxed for 3 h. The yellow powdery solid was collected by filtration, washed with CH_2Cl_2, acetone, and Et_2O, and dried in vacuo. Yield: 13.6 mg. (Immobilized ratio in PMO: 3% Pt/Si)

Synthesis of [Pt(ppy)(bpy)]PF_6 physisorbed on BPy-PMO (PtPF_6+PMO). PtPF_6+PMO was synthesized following the synthetic procedure for **PtCl + PMO**, in which [Pt(ppy)(bpy)]$(PF_6)_2$ was used (32.5 mg, 0.05 mmol) instead of [Pt(ppy)(bpy)]Cl. Yield: 13.6 mg. (Immobilized ratio in PMO: 2% Pt/Si)

Table S1 The XPS binding energy and the full-width at half-maximum of **Pt-PMO** and [Pt(ppy)(bpy)]Cl.

	**Pt-PMO**		[Pt(ppy)(bpy)]Cl	
	Pt $4f_{7/2}$	Pt $4f_{5/2}$	Pt $4f_{7/2}$	Pt $4f_{5/2}$
Binding energy / eV	72.1	75.1	72.1	75.3
Full-width at half-maximum / eV	2.25	2.25	1.80	1.80

Table S2 The Brunauer-Emmett-Teller (BET) surface area, all pore volume and Average pore diameter (nm) of BPy-PMO, **Pt-PMO**, and **Pt-PMO-R**. Average pore diameter (nm) is estimated by the non-linear density functional theory (NLDFT) analysis.

	BET / m^2/g	Pore volume / cm^3/g	Average pore diameter / nm
BPy-PMO	680	0.769	4.67
**Pt-PMO**	393	0.410	3.87
**Pt-PMO-R**	420	0.553	4.16

Table S3 Photophysical data for **Pt-PMO**, **Pt-PMO-R**, **Pt-PMO-LY**, **Pt-PMO-Y**, and [Pt(ppy)(bpy)]Cl.

	**Pt-PMO**	**Pt-PMO-R**	**Pt-PMO-LY**	**Pt-PMO-Y**	[Pt(ppy)(bpy)]Cl	
					Solid	Solution[f]
298 K						
λ_{em}[a] / nm	–	630	–	–	640	487, 519, 552
Φ[b]	<0.01	0.11	<0.01	<0.01	0.12	<0.01
τ[c] / ns	–	97.9	–	–	123	–
k_r[d] / s^{-1}	–	1.1×10^6	–	–	9.8×10^5	–
k_{nr}[e] / s^{-1}	–	9.1×10^6	–	–	7.2×10^6	–
77 K						
λ_{em}[a] / nm	554(sh), 642	670	481, 514, 552	618	675	483, 519, 551
Φ[b]	0.04	0.29	0.39	0.05	0.41	0.15

[a] Emission maximum wavelengths. [b] Emission quantum yields. [c] Emission lifetimes. [d] Radiative rate constants, $k_r = \Phi/\tau$. [e] Nonradiative rate constants, $k_{nr} = k_r(1-\Phi)/\Phi$. [f] In methanol (at 298 K) or a MeOH/EtOH = 1:1 (v:v) mixture (at 77 K).

Table S4 Crystal parameters and refinement data of $[Pt(ppy)(bpy)](PF_6)$.

T / K	93
Formula	$C_{21}H_{16}F_6N_3PPt$
Formula weight	650.43
Crystal system	Orthorhombic
Space group	*Pmmn* (#59)
a / Å	21.8291(6)
b / Å	15.6673(4)
c / Å	3.6048(1)
V / Å^3	1232.85(6)
Z	2
D_{cal} / g cm^{-3}	1.752
μ / mm^{-1}	11.791
Reflections collected	6302
Unique reflections	1347
R_{int}	0.0294
GOF	1.079
R_1 ($I > 2.00\sigma(I)$)^a	0.0288
wR_2^{b}	0.0766

^{a} $R_1 = \Sigma||F_o|-|F_c|| / \Sigma|F_o|$. ^{b} $wR_2 = [\Sigma w(F_o^2 - F_c^2)/ \Sigma w(F_o)^2]^{1/2}$, $w = [\sigma_c^2(F_o^2) + (xP)^2 + yP]^{-1}$, $P = (F_o^2 - 2F_c^2)/3$.

Table S5 Selected interatomic distances (Å) and angles (deg) for $[Pt(ppy)(bpy)](PF_6)$.

Distances / Å			
Pt1-N1	2.059(5)	Pt1-N2/C9	2.015(3)
Angles / deg			
N1-Pt1-N2/C9	72.33(15)	N1-Pt1-N2^a/C9^a	109.47(15)
N1-Pt1-N1^a	162.0(3)		

References

1. (a) Miskowski, V. M., Houlding, V. H., Che, C.-M. & Wang, Y. Electronic Spectra and Photophysics of Platinum(II) Complexes with α-Diimine Lligands. Mixed Complexes with Halide Ligands. *Inorg. Chem.* **32**, 2518-2524 (1993); (b) Kato, M., Kosuge, C., Morii, K., Ahn, J. S., Kitagawa, H., Mitani, T., Matsushita, M., Kato, T., Yano, S. & Kimura, M. Luminescence Properties and Crystal Structures of Dicyano(diimine)platinum(II) Complexes Controlled by Pt···Pt and π-π Interactions *Inorg. Chem.* **38**, 1638-1641 (1999).
2. Yoshida, M., Saito, K., Matsukawa, H., Yanagida, S., Ebina, M., Maegawa, Y., Inagaki, S., Kobayashi, A. & Kato, M. Immobilization of Luminescent Platinum(II) Complexes on Periodic Mesoporous Organosilica and their Water Reduction Photocatalysis. *J. Photochem. Photobiol. A: Chem.* **358**, 334-344 (2018).
3. Godbert, N., Pugliese, T., Aiello, I., Bellusci, A., Crispini, A. & Ghedini, M. Efficient, Ultrafast, Microwave-Assisted Syntheses of Cycloplatinated Complexes, *Eur. J. Inorg. Chem.* 5105-5111 (2007).
4. Araki, K., Fuse, M., Kishi, N., Shiraishi, S., Kodama, T. & Uchida, Y. Tris(6,6'-diamino-2,2'-bipyridine)ruthenium(II): Effect of High Interligand Steric Strain on the Structure, *Bull. Chem. Soc. Jpn.* **63**, 1299-1304 (1990).
